# Preoperative Posterior Paraspinal Fatty Infiltration and an Exploratory Axial Muscle Imaging Phenotype in Surgically Treated Metastatic Epidural Spinal Cord Compression: A Retrospective Cohort Study

**DOI:** 10.3390/medicina62091720

**Published:** 2026-09-07

**Authors:** Aydin Talat Baydar, Muhammed Bayindir, Bilal Yekeler, Mairambek Murtazaev, Baran Taskala

**Affiliations:** Department of Neurosurgery, Başakşehir Çam and Sakura City Hospital, Istanbul 34480, Turkey; muhammed.bayindir1@saglik.gov.tr (M.B.); bilalyekeler@gmail.com (B.Y.); mairambekmurtazaev723@gmail.com (M.M.); baran.taskala@saglik.gov.tr (B.T.)

**Keywords:** metastatic epidural spinal cord compression, spinal metastases, sarcopenia, myosteatosis, psoas, paraspinal muscle, Goutallier, early neurological improvement, survival

## Abstract

*Background and Objectives*: Prognostic assessment in metastatic epidural spinal cord compression (MESCC) requires estimation of survival, systemic condition, and the likelihood of early functional improvement. We evaluated whether posterior paraspinal fatty infiltration and an exploratory routine-imaging axial muscle phenotype derived from preoperative L3 CT/MRI were associated with overall survival and early neurological improvement after surgery for MESCC. *Materials and Methods*: Consecutive adults who underwent surgery for MESCC (Bilsky grade 1C–3) between June 2020 and November 2025 were retrospectively analyzed. Psoas quantity was measured on preoperative L3 computed tomography using the psoas-to-vertebral-body ratio (PVR) and psoas muscle index. Posterior paraspinal muscle quality was graded on axial T2-weighted L3 magnetic resonance imaging using a Goutallier-style scale in the multifidus/erector spinae compartment. The primary exposure was high-grade posterior paraspinal fatty infiltration, defined as patient-level maximum Goutallier grade 3–4. An exploratory secondary phenotype combined lower PVR by sex-specific cohort median with Goutallier grade 3–4. *Results*: Among 133 eligible patients, PVR measurements were available in 119, Goutallier grading in 112, and complete combined phenotyping in 109. Median survival was 383 days for Goutallier 0–2 and 144.5 days for Goutallier 3–4. In the primary clinical/Katagiri-adjusted Cox model, Goutallier 3–4 was associated with worse overall survival (hazard ratio [HR] 1.80, 95% confidence interval [CI] 1.06–3.07; *p* = 0.029). The exploratory both-abnormalities subgroup comprised 21 patients, with median survival of 73 days, and among patients with a preoperative Frankel deficit, early Frankel improvement occurred in 2/15 (13.3%); the adjusted overall-survival HR was 2.64 (95% CI 1.15–6.03; *p* = 0.021), whereas binary-outcome estimates were imprecise. *Conclusions*: High-grade posterior paraspinal fatty infiltration was associated with inferior survival and lower early neurological improvement after surgery for MESCC. The combined phenotype identified a small high-risk subgroup but should be regarded as hypothesis-generating. These routine-imaging markers require external validation and should not be used as stand-alone surgical selection criteria.

## 1. Introduction

Surgery for metastatic epidural spinal cord compression (MESCC) is generally considered when the expected neurological, mechanical, or oncological benefit is proportionate to the patient’s anticipated survival and systemic condition. Direct decompressive surgery can improve function in selected patients with MESCC [1], but contemporary decision-making has moved beyond decompression alone toward integrated frameworks that incorporate neurological compression, tumor radiosensitivity, mechanical instability, and systemic condition. The NOMS framework, the Spinal Instability Neoplastic Score (SINS), and the Bilsky epidural spinal cord compression scale provide a common language for several of these domains [2,3,4].

Survival estimation remains central to treatment proportionality. Prognostic systems such as Tomita, revised Tokuhashi, modified Bauer, Katagiri, and the New England Spinal Metastasis Score emphasize primary tumor biology, visceral disease, performance status, ambulatory status, and laboratory surrogates of systemic condition [5,6,7,8,9]. These tools are clinically useful, but they do not fully capture the patient’s biological capacity to tolerate surgery, mobilize after treatment, achieve functional improvement, and continue postoperative oncological therapy.

Body composition has emerged as a measurable imaging domain of systemic condition. L3-based computed tomography (CT) measurements are widely used in oncology, and muscle depletion has been associated with inferior survival across multiple cancer populations [10,11,12,13,14]. In metastatic spine surgery, psoas-derived morphometric indices have been associated with 90-day and overall mortality in prior cohorts [15,16,17,18]. However, psoas quantity is an incomplete surrogate for sarcopenia and may reflect sex, body habitus, nutritional status, inflammation, performance status, and tumor biology rather than a discrete spine-specific physiological reserve. The psoas muscle index may also be an imperfect proxy for whole-body skeletal muscle mass in cancer patients [19].

Posterior paraspinal muscle quality may be particularly relevant in spine oncology. The multifidus and erector spinae compartments contribute to posture, extension strength, segmental control, and mobilization after posterior spinal surgery. Fatty infiltration of these muscles may reflect chronic axial muscle degeneration, disuse, denervation, aging, or systemic cancer-related deterioration [20,21]. Although Goutallier-style grading is semiquantitative, it is feasible on routine magnetic resonance imaging (MRI) and has shown acceptable reliability in lumbar paraspinal assessment [22,23,24].

Recent spinal oncology literature has emphasized that frailty and sarcopenia tools remain heterogeneous and incompletely validated in this population [25]. Advanced MRI-derived techniques such as spinal tractography have also been explored as prognostic tools in spinal cord injury, although their routine role remains incompletely standardized [26]. Such work illustrates the broader interest in imaging-based prognostication, but it does not directly address systemic health status. This creates a practical gap: surgeons need feasible prognostic markers derived from routine preoperative imaging, but these markers should be interpreted cautiously and externally validated before incorporation into treatment algorithms. We therefore evaluated whether high-grade posterior paraspinal fatty infiltration was associated with survival and early neurological improvement after surgery for MESCC. The primary exposure was patient-level maximum posterior paraspinal Goutallier grade 3–4. A combined axial muscle phenotype integrating lower PVR by sex-specific cohort median and Goutallier grade 3–4 was evaluated as an exploratory secondary exposure.

## 2. Materials and Methods

### 2.1. Study Design, Setting, and Ethics

This retrospective cohort study included consecutive adult patients who underwent surgery for metastatic epidural spinal cord compression (MESCC) at Basaksehir Cam and Sakura City Hospital, a tertiary referral center, between June 2020 and November 2025. Clinical, radiological, operative, laboratory, and survival data were extracted from institutional records. Vital status was updated and administratively censored on 24 June 2026. The study was conducted in accordance with the Declaration of Helsinki and was approved by the Basaksehir Cam and Sakura City Hospital Ethics Committee on 23 December 2025 (approval number 425). The requirement for individual informed consent was waived because of the retrospective observational design.

### 2.2. Patient Selection

During the study period, 143 surgically treated spinal metastasis cases were screened. Nine patients with intradural metastases were excluded a priori because their biological behavior, operative indications, imaging measurement considerations, and postoperative recovery patterns differ from metastatic epidural disease. One pediatric patient was excluded because the study was restricted to adults. The final analytic cohort therefore included 133 adult patients who underwent surgery for MESCC, with preoperative Bilsky grades ranging from 1C to 3, and had sufficient clinical and survival data for analysis. For patients who underwent more than one metastatic spine operation during the study period, the first eligible operation was considered the index surgery unless otherwise specified. Cases were not excluded because of primary tumor type, spinal region, extent of surgery, or postoperative oncological treatment.

### 2.3. Imaging Timing and Selection

Preoperative CT and MRI examinations obtained within one month before surgery were used for body-composition assessment. When multiple eligible examinations were available, the study closest to the index surgery was selected. This approach was chosen to minimize misclassification from interval systemic decline, cancer progression, or treatment-related change while preserving the use of routine preoperative imaging.

### 2.4. Psoas Quantity Measurements

**Figure 1:**
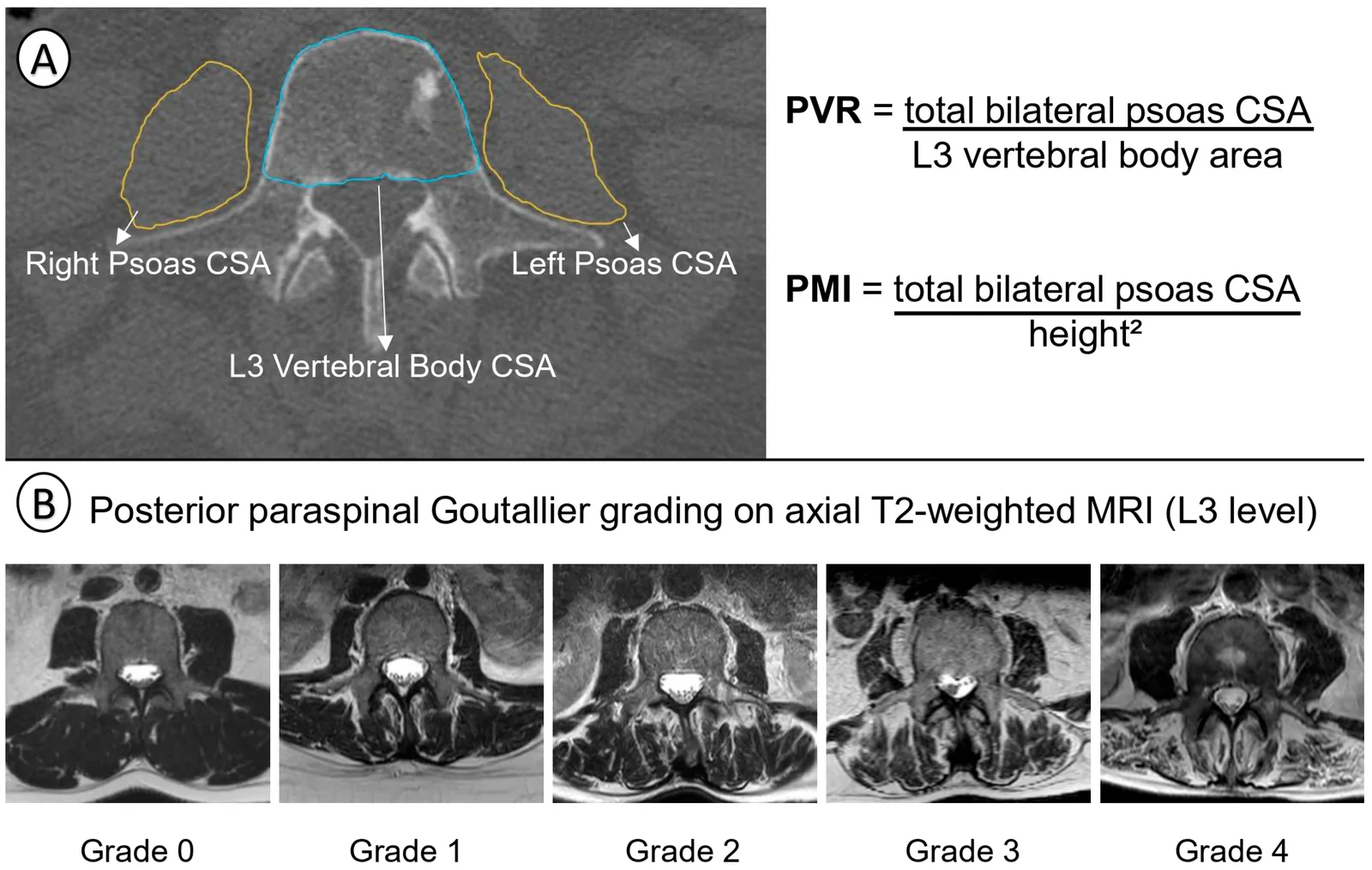
Imaging assessment of posterior paraspinal fatty infiltration and the exploratory combined axial muscle phenotype. (**A**) Example of L3 CT-based psoas assessment showing bilateral psoas cross-sectional area and L3 vertebral body cross-sectional area measurements, used to calculate the psoas-to-vertebral-body ratio and psoas muscle index. (**B**) Representative axial T2-weighted L3 MRI examples of posterior paraspinal Goutallier grades 0–4 in the multifidus/erector spinae compartment. For primary analyses, patient-level maximum posterior paraspinal Goutallier grade was dichotomized as low-grade fatty infiltration, grades 0–2, versus high-grade fatty infiltration, grades 3–4. CSA, cross-sectional area; MRI, magnetic resonance imaging; PVR, psoas-to-vertebral-body ratio; PMI, psoas muscle index.

Psoas measurements were performed on preoperative CT at the L3 level using the axial slice where both transverse processes were aligned (Figure 1A). Bilateral psoas cross-sectional areas and L3 vertebral body cross-sectional area were measured. The psoas-to-vertebral-body ratio (PVR) was calculated as total bilateral psoas cross-sectional area divided by L3 vertebral body area. The psoas muscle index (PMI) was calculated as total bilateral psoas area divided by height squared. Psoas radiodensity was recorded in Hounsfield units when CT quality permitted. PVR was selected as the main psoas quantity measure because it normalizes psoas area to patient-specific skeletal size and avoids reliance on external population cutoffs. PMI and psoas radiodensity were analyzed as supporting body-composition variables.

### 2.5. Posterior Paraspinal Fatty Infiltration Assessment

Posterior paraspinal fatty infiltration was graded on preoperative axial T2-weighted MRI at L3 (Figure 1B). The posterior paraspinal compartment included the multifidus and erector spinae muscles. For each side, the recorded grade represented the worst fatty infiltration observed in this combined posterior paraspinal compartment; multifidus and erector spinae grades were not recorded separately. The patient-level maximum of the right and left grades was used for primary analyses.

Axial T2-weighted imaging was selected because it was the most consistently available axial lumbar sequence across routine preoperative spine MRI protocols and provided reproducible visualization of the posterior paraspinal compartment at L3. The purpose of grading was semiquantitative classification of MRI-visible fatty infiltration on clinically available imaging rather than quantitative intramuscular fat measurement. Fatty infiltration was interpreted from the relative high-signal replacement of normal muscle architecture together with chronic compartmental morphology. This pragmatic method does not replace Dixon, chemical-shift, or other quantitative MRI fat-fraction techniques and should be interpreted as an imaging grade rather than a direct biological measurement of muscle fat content.

Goutallier-style grades were defined as follows: grade 0, no visible fatty infiltration; grade 1, minor fatty streaks; grade 2, fatty infiltration less than muscle; grade 3, fatty infiltration approximately equal to muscle; and grade 4, fatty infiltration greater than muscle. High-grade posterior paraspinal fatty infiltration was defined as patient-level maximum Goutallier grade 3 or 4.

### 2.6. Rationale for L3 Measurement

L3 was selected because it is the most commonly used cross-sectional body-composition level in oncology and allowed paired assessment of anterior axial muscle quantity and posterior paraspinal muscle quality [11,12]. This level also provides a pragmatic compromise between standard oncological body-composition methodology and assessment of the axial musculature. By avoiding the lower lumbosacral segments, the measurement was intended to reduce the influence of advanced L4-S1 degenerative stenosis, segmental instability, lumbosacral postoperative change, and focal fatty degeneration. Accordingly, L3 posterior paraspinal grading was interpreted as a routine-imaging axial muscle-quality marker rather than a direct measurement of the operated segment or a validated measure of spine-specific physiological reserve.

### 2.7. Measurement Availability and Inter-Rater Reliability

Cases were considered technically non-measurable when no usable L3-level imaging was available within the preoperative window or when tumor-related destruction, severe artifact, or anatomic distortion invalidated contouring or grading. Small non-distorting L3 lesions were retained and flagged. Transitional anatomy was retained when vertebral counting and measurement were reproducible. Body-composition exposures were not imputed because absent L3 imaging or anatomy-destroying tumors represented structural non-measurability rather than ordinary covariate missingness.

Posterior paraspinal Goutallier grading was performed by observers blinded to survival status and postoperative neurological outcomes. Inter-rater reliability was assessed in 40 randomly selected patients independently graded by two observers. Because Goutallier grading is ordinal, quadratic weighted Cohen’s kappa was selected as the primary reliability statistic. Exact agreement and within-one-grade agreement were also reported.

### 2.8. Exposures and Phenotype Definitions

The primary exposure was high-grade posterior paraspinal fatty infiltration, defined as patient-level maximum posterior paraspinal Goutallier grade 3–4. Psoas quantity was evaluated using PVR and PMI as continuous sex-standardized variables and as sex-specific median-based groups for visualization and exploratory phenotype construction.

The exploratory secondary exposure was a combined axial muscle phenotype defined among patients with both PVR and Goutallier data: (1) neither abnormality, defined as higher-PVR stratum and Goutallier grade 0–2; (2) one abnormality, defined as either lower-PVR stratum or Goutallier grade 3–4; and (3) both abnormalities, defined as lower-PVR stratum and Goutallier grade 3–4. The lower-PVR stratum was defined using sex-specific cohort medians. Because externally validated PVR thresholds for MESCC are not established, these sex-specific medians were used only to construct an internal exploratory phenotype and should not be interpreted as diagnostic sarcopenia cutoffs. The phenotype was intended to test two complementary routine-imaging features—anterior axial muscle quantity and posterior paraspinal muscle quality—and was not considered a validated measure of physiological reserve.

### 2.9. Tumor Biology and Clinical Covariates

Primary tumor biology was grouped using a Katagiri-style growth classification as slow, moderate, or rapid [8,27]. Slow tumors included hematologic/myeloma/lymphoma, breast, prostate, differentiated thyroid, and testis/germ-cell tumors. Moderate tumors included renal cell carcinoma, gynecologic tumors, sarcoma, and selected intermediate other solid tumors. Rapid tumors included lung cancer, colorectal/rectal, gastric, pancreatic/pancreatobiliary, hepatobiliary/hepatocellular carcinoma, melanoma, adrenal carcinoma, poorly differentiated neuroendocrine carcinoma, laryngeal/head-and-neck carcinoma, and anaplastic or mixed aggressive thyroid cancer. A four-group sensitivity classification was also used: hematologic/myeloma/lymphoma, favorable solid tumors, lung cancer, and other solid tumors. The full primary-pathology mapping is provided in Supplementary Table S1.

Clinical covariates included age, sex, Eastern Cooperative Oncology Group (ECOG) performance status, albumin, Bilsky grade 3 compression, preoperative Frankel grade, ambulatory status, SINS, surgical strategy, instrumentation, operative time, transfusion, neutrophil-to-lymphocyte ratio (NLR), and prognostic nutritional index (PNI). ECOG was recorded on the 0–4 scale, was available for all 133 patients, and was modeled as an ordinal covariate per one-grade increase in the regression models. Karnofsky Performance Status was not routinely documented and was not retrospectively derived from ECOG. NLR was log-transformed for regression modeling because of right-skewed distribution.

### 2.10. Surgical Heterogeneity, Postoperative Oncological Treatment, and Complication Adjudication

Surgical strategy was grouped into four broad categories: decompression-only or decompression with vertebroplasty, decompression with short instrumentation, decompression with long instrumentation, and corpectomy or 360-degree reconstruction. Surgical strategy was summarized descriptively by muscle phenotype. Surgical strategy was not included in the primary survival model because operative strategy is partly determined by tumor anatomy, instability, neurological compression, and patient condition and may lie on the treatment pathway rather than represent a purely baseline prognostic domain. A sensitivity model adding broad surgical strategy to the clinical tumor-adjusted model was performed.

Postoperative radiotherapy and systemic therapy within 90 days were collected descriptively. Systemic therapy represented tumor-specific oncological treatment and, where documented, included cytotoxic chemotherapy, immunotherapy, targeted or endocrine therapy, and hematologic/plasma-cell-directed regimens. Because receipt of postoperative oncological treatment depends partly on early survival, wound recovery, functional recovery, treatment eligibility, access to treatment, and cancer trajectory, these variables were not used as baseline adjustment covariates. Recorded complications and reoperations were manually adjudicated from the structured and free-text clinical fields. Planned staged procedures, procedures for a new metastatic level, and clearly preoperative events were excluded from index-related unplanned reoperation counts. Complications were summarized descriptively as any recorded perioperative/postoperative complication, wound-related complication, infectious complication, venous thromboembolism, and early unplanned index-related reoperation. Because exact onset dates were not uniformly available for all complication types, no formal comparative testing was performed for these adjudicated postoperative variables.

### 2.11. Outcomes

The primary outcome was overall survival from surgery to death or administrative censoring on 24 June 2026. Secondary survival outcomes included 90-day mortality, 365-day survival, and 365-day restricted mean survival time (RMST). Early neurological improvement was assessed using Frankel grade at postoperative day 10–14. Frankel improvement was defined as at least one-grade improvement from the preoperative Frankel grade. Patients with preoperative Frankel grade E were excluded from Frankel-improvement analyses because of ceiling effect. Postoperative ambulatory status and conversion to Frankel D/E among patients who were nonambulatory preoperatively were also summarized at the same early assessment. Length of stay, in-hospital mortality, recorded complications, reoperations, and receipt of postoperative oncological treatment were reported descriptively. No patient died before the scheduled postoperative day 10–14 neurological assessment.

### 2.12. Statistical Analysis

Continuous variables are reported as median and interquartile range, and categorical variables are reported as counts and percentages. Between-group comparisons were descriptive and were not used for variable selection. Overall survival was calculated from the date of surgery to death or administrative censoring on 24 June 2026. Follow-up duration was summarized using the reverse Kaplan–Meier method.

Kaplan–Meier curves and log-rank tests were used for unadjusted survival comparisons. Restricted mean survival time (RMST) was calculated to 365 days to provide an absolute survival-time contrast at a clinically relevant time horizon [28]. Cox proportional hazards regression was used for adjusted survival analyses. The primary inferential model evaluated the association between Goutallier grade 3–4 and overall survival after adjustment for age, sex, ECOG performance status, albumin, Bilsky grade 3 compression, and Katagiri-style tumor-growth group. The proportional-hazards assumption was evaluated using Schoenfeld residual-based tests for the exposure term and global model where available in each Cox model.

The exploratory combined axial muscle phenotype, psoas-only measures, ordinal Goutallier grade, alternative inflammatory-nutritional adjustment, four-group tumor-biology adjustment, solid-tumor-only analysis, and surgical-strategy-adjusted analysis were considered secondary or sensitivity analyses. The alternative inflammatory-nutritional model replaced albumin with PNI and added log-transformed NLR while retaining age, sex, ECOG performance status, Bilsky grade 3 compression, and Katagiri-style tumor-growth group. Albumin and PNI were not included in the same model because PNI incorporates albumin. PNI was modeled per 5-point decrease to improve interpretability.

Firth bias-reduced logistic regression was used for secondary binary outcomes because of limited event counts. The fully adjusted 90-day mortality Firth models included age, sex, ECOG performance status, albumin, Bilsky grade 3 compression, and Katagiri-style tumor-growth group. The fully adjusted early Frankel-improvement models included patients with preoperative Frankel deficit and adjusted for age, sex, ECOG performance status, Bilsky grade 3 compression, Katagiri-style tumor-growth group, and preoperative Frankel grade. Because these secondary binary-outcome analyses had limited events, particularly within the 21-patient both-abnormalities subgroup, they were interpreted as exploratory effect-size analyses rather than prediction models.

No adjustment for multiplicity was performed; therefore, secondary and sensitivity analyses should be interpreted as exploratory. All analyses were performed using R software (R Foundation for Statistical Computing, Vienna, Austria; version 4.6.0) with the survival, logistf, survRM2, tableone, tidyverse, and ggplot2 packages. A two-sided *p* value < 0.05 was considered statistically significant.

### 2.13. Use of Generative Artificial Intelligence

Generative artificial intelligence (ChatGPT v5.6 Sol; OpenAI, San Francisco, CA, USA) was used to assist with language editing, structural refinement, and manuscript-style revision. The tool was not used for data collection, image measurements, statistical analysis, or generation of primary results. All AI-assisted text was critically reviewed and edited by the authors, who take full responsibility for the accuracy, integrity, and final content of the manuscript.

## 3. Results

### 3.1. Cohort and Measurement Availability

Of 143 surgically treated spinal metastasis cases screened during the study period, nine intradural metastases and one pediatric case were excluded, leaving 133 adult patients who underwent surgery for MESCC with Bilsky grade 1C–3 in the final analytic cohort. Survival status was administratively censored on 24 June 2026. Median follow-up by the reverse Kaplan–Meier method was 687 days (95% CI, 482–826) in the full cohort and 687 days (95% CI, 478–826) among patients with posterior paraspinal Goutallier grading.

PVR, PMI, and psoas radiodensity measurements were available in 119 patients (89.5%). Posterior paraspinal Goutallier grading was available in 112 patients (84.2%), and complete combined axial muscle phenotyping was available in 109 patients (82.0%). Baseline comparisons according to posterior paraspinal Goutallier measurability and complete phenotype availability are provided in Supplementary Table S2A,B.

ECOG performance status was available for all 133 patients: ECOG 0 in two (1.5%), ECOG 1 in 48 (36.1%), ECOG 2 in 34 (25.6%), ECOG 3 in 24 (18.0%), and ECOG 4 in 25 (18.8%). Patients without measurable Goutallier data did not differ significantly from measurable patients in age, ECOG, albumin, sex, Bilsky grade 3, preoperative Frankel deficit, primary tumor group, Katagiri-style growth group, or observed survival (all descriptive *p* > 0.05). Likewise, patients with incomplete versus complete combined-phenotype data showed no statistically significant differences across these measured domains (Supplementary Table S2A,B). These comparisons reduce concern for major measured selection differences but do not exclude bias related to unmeasured disease burden or imaging availability.

### 3.2. Baseline Characteristics According to Goutallier Grade

Among the 112 patients with posterior paraspinal Goutallier grading, 74 (66.1%) had Goutallier grade 0–2, and 38 (33.9%) had Goutallier grade 3–4. Baseline characteristics according to Goutallier group are summarized in Table 1. Patients with Goutallier grade 3–4 were older (median 68.5 vs. 61.0 years; *p* = 0.005), had worse ECOG performance status (*p* = 0.009), lower albumin (median 3.35 vs. 3.85 g/dL; *p* = 0.006), lower PNI (median 33.8 vs. 39.3; *p* = 0.001), higher NLR (*p* = 0.036), and a higher transfusion rate (76.3% vs. 48.6%; *p* = 0.005). The full categorical ECOG distribution is shown in Table 1. Primary tumor group and Katagiri-style tumor-growth distribution are also shown in Table 1.

### 3.3. Imaging Distribution and Inter-Rater Reliability

Among patients with measurable posterior paraspinal Goutallier grade, patient-level maximum grades were distributed as follows: grade 0 in six patients (5.4%), grade 1 in 24 (21.4%), grade 2 in 44 (39.3%), grade 3 in 30 (26.8%), and grade 4 in eight (7.1%). The lower-PVR stratum, defined by sex-specific cohort median, was present in 59 of 119 patients (49.6%). Among the 109 patients with complete PVR and Goutallier data, 39 (35.8%) had neither abnormality, 49 (45.0%) had one abnormality, and 21 (19.3%) had both the lower-PVR stratum and Goutallier grade 3–4 (Table 2).

Inter-rater reliability for patient-level maximum Goutallier grade was assessed in 40 randomly selected patients. Exact agreement was 80.0%, within-one-grade agreement was 100.0%, and quadratic weighted kappa was 0.887. Side-specific weighted kappa values were 0.907 for the right side and 0.874 for the left side (Table 2).

### 3.4. Tumor Biology, Performance Status, Surgical Strategy, Postoperative Treatment, and Outcomes by Exploratory Combined Phenotype

Tumor-biology group, Katagiri-style tumor-growth group, ECOG performance status, surgical strategy, transfusion, postoperative treatment, and clinical outcomes according to the exploratory combined axial muscle phenotype are summarized in Table 3. ECOG shifted toward poorer categories across the phenotype groups (Kruskal–Wallis *p* = 0.007): ECOG 4 was present in 7.7% of patients with neither abnormality, 18.4% with one abnormality, and 42.9% with both abnormalities. Transfusion was recorded in 35.9%, 65.3%, and 81.0% of these groups, respectively. Median postoperative length of stay was 4, 5, and 7 days. Any recorded perioperative/postoperative complication occurred in 33.3%, 30.6%, and 47.6%; infectious complications in 2.6%, 6.1%, and 19.0%; venous thromboembolism in 5.1%, 0%, and 9.5%; and early unplanned index-related reoperation in 12.8%, 16.3%, and 19.0%, respectively. In-hospital mortality was 0%, 2.0%, and 4.8%. Postoperative systemic therapy within 90 days was recorded in 69.2%, 53.1%, and 23.8%, and postoperative radiotherapy within 90 days in 25.6%, 36.7%, and 14.3%, respectively. At postoperative day 10–14, ambulatory Frankel D/E status was present in 92.3%, 75.5%, and 61.9%; among patients nonambulatory preoperatively, ambulatory conversion occurred in 66.7%, 35.3%, and 0%, respectively. Postoperative treatment and complication comparisons were descriptive and were not formally tested because of survival-to-treatment, follow-up, timing, and denominator considerations.

### 3.5. Unadjusted Survival and Absolute Survival-Time Differences

**Figure 2:**
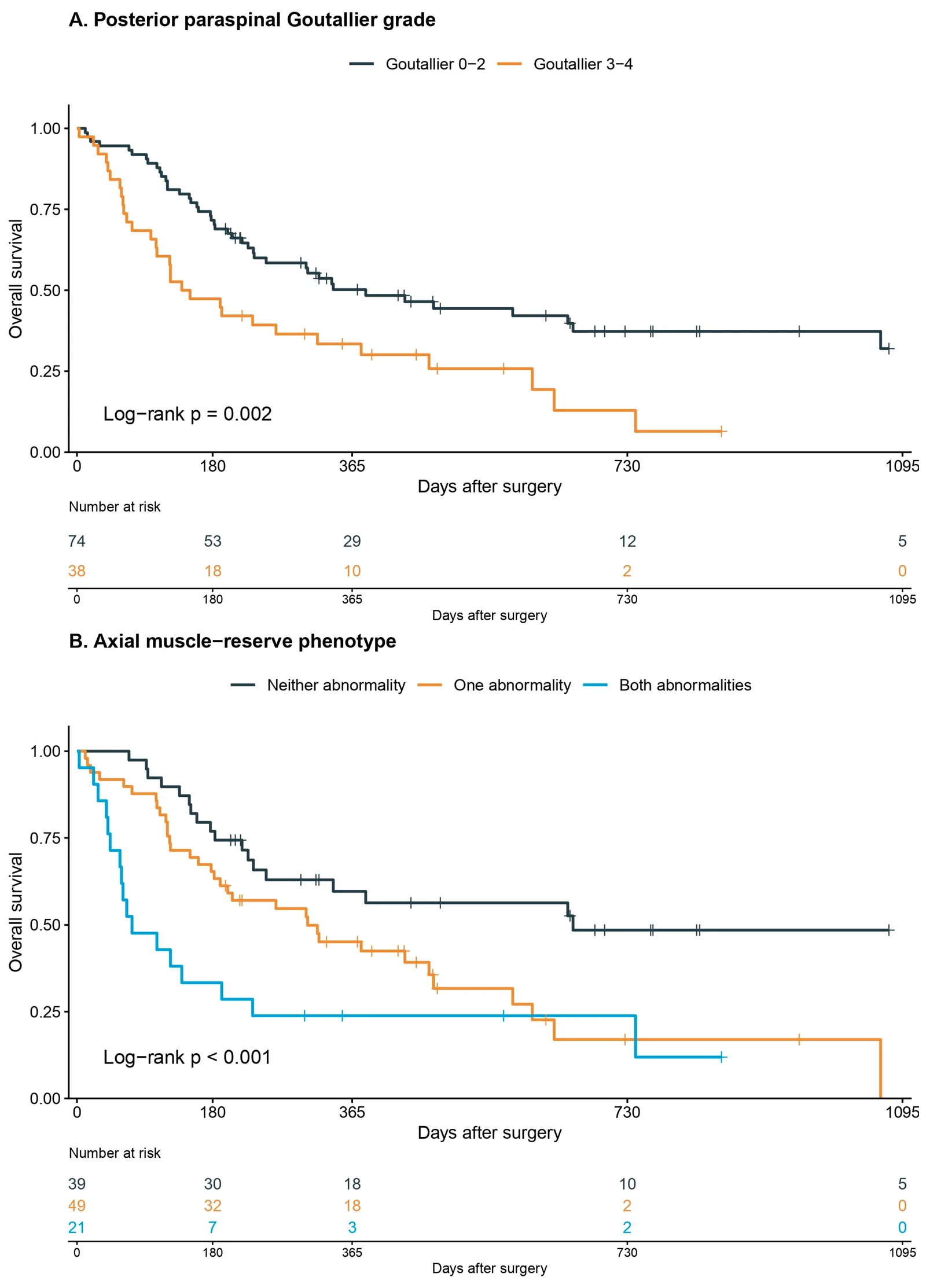
Overall survival according to posterior paraspinal Goutallier grade and the exploratory combined axial muscle phenotype. (**A**) Kaplan–Meier survival curves stratified by patient-level maximum posterior paraspinal Goutallier grade 0–2 versus 3–4. (**B**) Kaplan–Meier survival curves stratified by the combined phenotype: neither abnormality, one abnormality, or both the lower-PVR stratum and Goutallier grade 3–4. Number-at-risk tables are shown beneath each panel. Kaplan–Meier curves are unadjusted; adjusted associations are presented in Table 4 and Figure 3. The label ‘axial muscle-reserve phenotype’ within Panel (**B**) refers to the exploratory combined axial muscle phenotype defined in the present study and should not be interpreted as a validated physiological reserve measure.

Among the 112 patients with posterior paraspinal Goutallier grading, 72 deaths were observed by the administrative censoring date. Median survival was 383 days for Goutallier grade 0–2 and 144.5 days for Goutallier grade 3–4 (log-rank *p* = 0.002) (Figure 2A). Kaplan–Meier-estimated 365-day survival was 50.2% (95% CI 39.6–63.6) for Goutallier 0–2 and 33.5% (95% CI 21.2–52.8) for Goutallier 3–4. The 365-day RMST was 268.0 days for Goutallier 0–2 and 195.9 days for Goutallier 3–4, corresponding to a 72.1-day difference (95% CI −123.2 to −21.0; *p* = 0.006) (Supplementary Table S3A,B).

**Figure 3:**
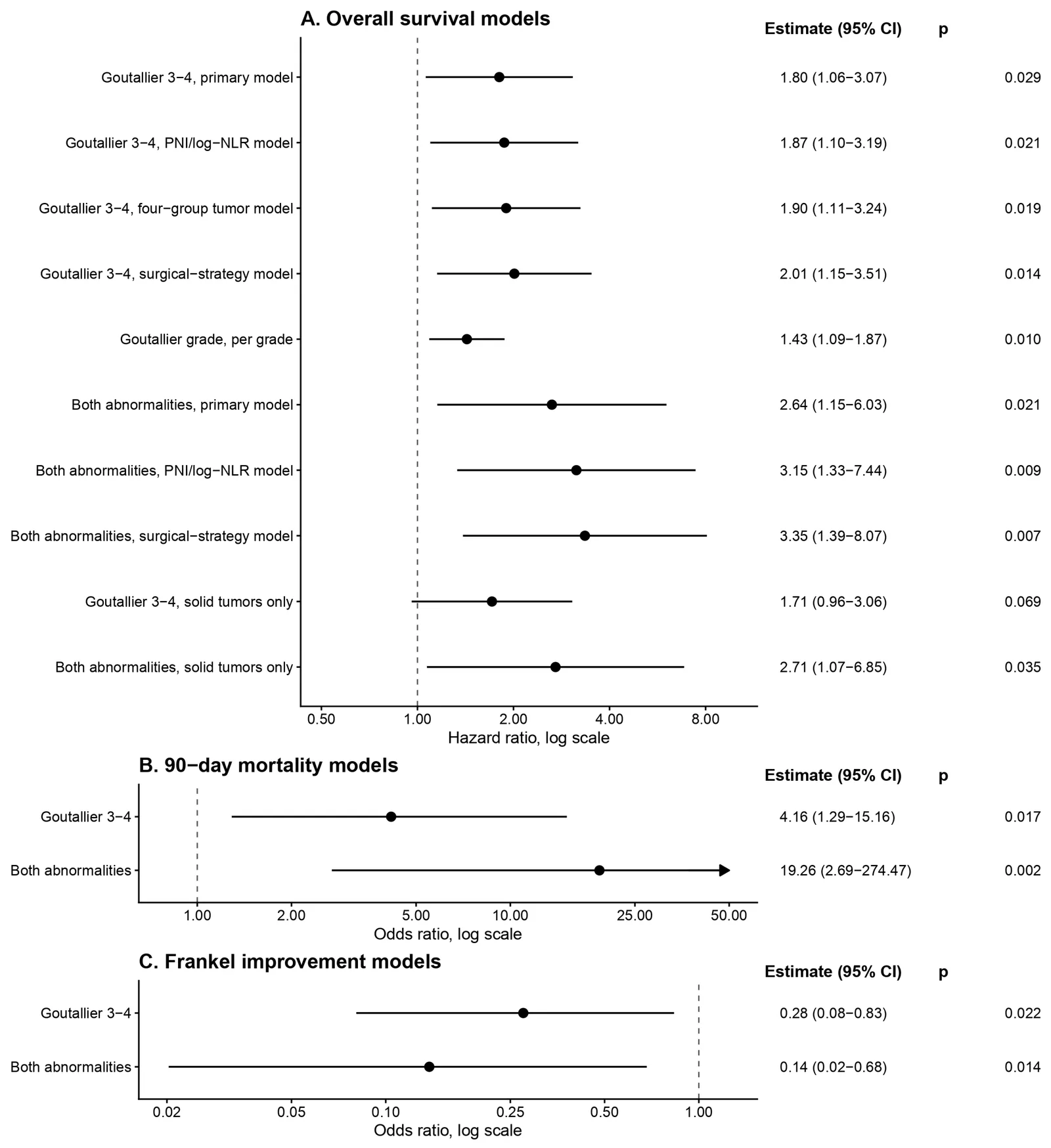
Main and sensitivity models for survival, 90-day mortality, and early neurological improvement. Forest plots show exposure estimates with 95% confidence intervals. Panel (**A**) reports hazard ratios from Cox proportional hazards models for overall survival. Panel (**B**) reports odds ratios from adjusted Firth logistic regression models for 90-day mortality. Panel (**C**) reports odds ratios from adjusted Firth logistic regression models for early Frankel improvement among patients with preoperative Frankel deficit. Arrows indicate confidence intervals extending beyond the plotted axis. Numerical estimates and *p* values are printed to the right of each panel. Combined-phenotype estimates are exploratory because of the small both-abnormalities subgroup. CI, confidence interval; PNI, prognostic nutritional index; NLR, neutrophil-to-lymphocyte ratio.

Among the 109 patients with complete phenotype data, 69 deaths were observed by the administrative censoring date. Median survival was 658 days for patients with neither abnormality, 306 days for those with one abnormality, and 73 days for the 21 patients with both the lower-PVR stratum and Goutallier grade 3–4 (log-rank *p* < 0.001) (Figure 2B). Kaplan–Meier-estimated 365-day survival was 59.6%, 45.1%, and 23.8%, respectively. Compared with the neither-abnormality group, the both-abnormalities group had a 141.2-day lower 365-day RMST (95% CI −206.5 to −75.8; *p* < 0.001) (Table 3; Supplementary Table S3A,B). These unadjusted survival curves are descriptive; adjusted associations are reported in Table 4 and Figure 3.

### 3.6. Adjusted Overall Survival Models

In the primary clinical/Katagiri-adjusted Cox model, the HR for Goutallier grade 3–4 versus grade 0–2 was 1.80 (95% CI 1.06–3.07; *p* = 0.029). The corresponding HRs were 1.87 (95% CI 1.10–3.19; *p* = 0.021) in the PNI/log-NLR model, 1.90 (95% CI 1.11–3.24; *p* = 0.019) in the four-group tumor-biology model, and 2.01 (95% CI 1.15–3.51; *p* = 0.014) in the surgical-strategy sensitivity model. In the ordinal model, each one-grade increase in Goutallier score had an HR of 1.43 (95% CI 1.09–1.87; *p* = 0.010) (Table 4; Figure 3A).

For psoas-only quantity measures, the lower-PVR stratum had an adjusted HR of 1.24 (95% CI 0.73–2.10; *p* = 0.424), and the lower-PMI stratum had an adjusted HR of 1.28 (95% CI 0.76–2.15; *p* = 0.347). Continuous sex-standardized PVR, PMI, and psoas radiodensity models are provided in Supplementary Table S4.

In the exploratory combined-phenotype model, compared with patients with neither abnormality, the HR was 1.23 (95% CI 0.60–2.55; *p* = 0.574) for one abnormality and 2.64 (95% CI 1.15–6.03; *p* = 0.021) for both abnormalities. The HR for both abnormalities was 3.15 (95% CI 1.33–7.44; *p* = 0.009) in the PNI/log-NLR model and 3.35 (95% CI 1.39–8.07; *p* = 0.007) in the surgical-strategy sensitivity model (Table 4; Figure 3A). These estimates were interpreted as exploratory because the both-abnormalities group contained only 21 patients, and the phenotype used an internally derived PVR threshold.

In the solid-tumor-only sensitivity analysis, the HR for Goutallier grade 3–4 was 1.71 (95% CI 0.96–3.06; *p* = 0.069), and the HR for both abnormalities was 2.71 (95% CI 1.07–6.85; *p* = 0.035). Schoenfeld residual-based tests for the exposure terms and global models are provided in Supplementary Table S5. Full multivariable Cox model coefficients, including all adjustment covariates, are provided in Supplementary Table S6.

### 3.7. Ninety-Day Mortality and Early Neurological Improvement

Ninety-day mortality was 8.1% in the Goutallier 0–2 group and 31.6% in the Goutallier 3–4 group. In adjusted Firth logistic regression, the OR for Goutallier grade 3–4 was 4.16 (95% CI 1.29–15.16; *p* = 0.017). For the exploratory combined phenotype, the OR for both abnormalities versus neither abnormality was 19.26 (95% CI 2.69–274.47; *p* = 0.002) (Table 4; Figure 3B). The very wide confidence interval and small 21-patient both-abnormalities subgroup indicate substantial imprecision; this estimate should be regarded as a hypothesis-generating signal rather than a clinically operative risk threshold.

Among patients with a preoperative Frankel deficit, early Frankel improvement by postoperative day 10–14 occurred in 25 of 39 patients (64.1%) with Goutallier 0–2 and seven of 27 patients (25.9%) with Goutallier 3–4. By combined phenotype, early Frankel improvement occurred in 17 of 24 patients (70.8%) with neither abnormality, 12 of 24 (50.0%) with one abnormality, and two of 15 (13.3%) with both abnormalities (Table 3). In adjusted Firth logistic regression, the OR for Goutallier grade 3–4 was 0.28 (95% CI 0.08–0.83; *p* = 0.022), and the OR for both abnormalities versus neither abnormality was 0.14 (95% CI 0.02–0.68; *p* = 0.014) (Table 4; Figure 3C). Full Firth logistic regression model coefficients, including all adjustment covariates, are provided in Supplementary Table S7. These results concern early neurological improvement only and do not establish durable functional recovery.

## 4. Discussion

In this retrospective cohort of patients undergoing surgery for MESCC, high-grade posterior paraspinal fatty infiltration was associated with inferior overall survival after adjustment for clinical status and tumor biology. In contrast, psoas quantity separated crude survival but was not associated with survival after multivariable adjustment. The exploratory combined axial muscle phenotype identified a small subgroup with short median survival, high 90-day mortality, and low early Frankel improvement. The principal finding is therefore the adjusted association of posterior paraspinal Goutallier grade with survival; the combined phenotype should be viewed as a secondary hypothesis-generating signal.

The present findings align with, but also refine, the existing spinal oncology sarcopenia literature. Bourassa-Moreau et al. reported that sarcopenia, rather than frailty, predicted early mortality and adverse events after emergent surgery for metastatic spine disease [15]. Zakaria et al. similarly found that psoas-derived sarcopenia predicted 90-day and overall mortality in patients undergoing surgery for spinal metastases [16], and Hu et al. reported that decreased psoas muscle area was associated with 90-day and 1-year survival after spinal metastasis surgery [17]. These studies established the clinical relevance of morphometric muscle assessment in metastatic spine surgery. Our study differs in two important ways: first, psoas quantity alone did not retain an adjusted association with survival in our cohort; second, posterior paraspinal fatty infiltration remained associated with overall survival after adjustment, suggesting that muscle quality and anatomical compartment may provide information not captured by simple psoas size.

This divergence is biologically plausible but should not be interpreted mechanistically. Pielkenrood et al. prospectively evaluated patients with spinal metastases receiving radiotherapy and found that muscle density, rather than sarcopenia or body fat distribution, was independently associated with 90- and 365-day survival [29]. Bongers et al. also examined CT-derived body composition in surgically treated spinal metastases and placed psoas, paraspinal, and other body-composition variables within a broader imaging-biomarker framework [18]. Taken together with our results, the available literature supports further study of muscle quality, attenuation, and MRI-visible fatty infiltration as prognostic imaging features beyond isolated psoas area.

The psoas-specific result should not be interpreted as evidence that psoas measurements are useless. Rather, psoas area appears to be a convenient but nonspecific marker. Psoas measurements are easy to obtain, have been used in several metastatic spine and skeletal oncology cohorts [15,16,17,18,30], and may be useful for broad risk stratification. However, Pigneur et al. showed that PMI is not a reliable substitute for whole-L3 skeletal muscle index in cancer patients [19]. This limitation is directly relevant to spinal oncology because psoas size may reflect body habitus, sex, systemic inflammation, cachexia, and tumor biology while failing to characterize posterior extensor degeneration or other aspects of axial muscle quality.

Posterior paraspinal fatty infiltration may represent a different imaging dimension. The multifidus and erector spinae compartments are central to posture, extension, segmental control, and mobilization after spine surgery. Fatty infiltration of these muscles may reflect chronic axial muscle degeneration, disuse, denervation, cancer-related cachexia, aging, or a combination of these processes. In our cohort, high-grade fatty infiltration also clustered with older age, worse ECOG performance status, lower albumin and PNI, and higher NLR. Accordingly, the observed association may partly reflect global frailty, nutritional–inflammatory status, disease burden, and overall systemic condition rather than a distinct spine-specific biological reserve. Adjustment for measured covariates reduced this concern but cannot eliminate residual confounding.

The paraspinal muscle literature outside oncology supports cautious use of this marker. In degenerative lumbar disease, Goutallier-style paraspinal grading has shown acceptable reliability and associations with functional or perioperative outcomes [22,23,24]. More recently, Kulkarni et al. reported that lumbar paraspinal Goutallier grade correlated with frailty and predicted 180-day complications, reoperation, and non-home discharge after elective lumbar surgery [31]. Although that population differs from MESCC, it reinforces the concept that posterior paraspinal fatty infiltration may serve as an accessible imaging surrogate of reduced systemic and functional condition. Importantly, reproducibility does not establish biological validity: our high inter-rater agreement supports reliable grading within this cohort but does not validate Goutallier grade against Dixon MRI, quantitative fat fraction, or histological muscle composition.

The anatomical distinction is important. The exploratory combined phenotype integrates anterior axial muscle quantity, measured by psoas-derived indices, with posterior paraspinal muscle quality, assessed in the multifidus/erector spinae compartment. It should not be interpreted as a psoas-only sarcopenia measure or as formal sarcopenia because muscle strength, gait speed, handgrip strength, and physical performance were not measured. It is better considered an internally derived routine-imaging axial muscle phenotype.

The L3 level was selected for pragmatic and conceptual reasons. It is widely used in oncology body-composition assessment and permits paired evaluation of psoas quantity and posterior paraspinal quality [11,12]. Measuring at L3 was also intended to reduce the influence of severe lower lumbosacral degenerative disease. Nevertheless, L3 is not the operated segment for many thoracic or cervical MESCC cases, and lumbar fatty infiltration may also reflect age, chronic pain, disuse, stenosis, or degeneration. L3 posterior paraspinal grading should therefore be interpreted as an axial muscle-quality marker derived from routine imaging rather than a direct local measure of the surgical segment.

The absolute differences observed across the combined phenotype groups are clinically interesting but require restraint. The both-abnormalities group contained only 21 patients and had a median survival of 73 days, 90-day mortality of 52.4%, Kaplan–Meier-estimated 1-year survival of 23.8%, and a 141.2-day reduction in 365-day RMST compared with patients with neither abnormality. The adjusted 90-day mortality OR of 19.26 had a very wide 95% CI of 2.69–274.47, and the lower-PVR threshold was defined by sex-specific medians internal to this cohort. These findings should therefore be presented as an exploratory signal rather than a clinically operative phenotype or transportable cutoff. The study does not show that surgical decisions should have changed, nor does it demonstrate that a different operation would have improved outcomes.

The early neurological findings are also relevant to surgical counseling. Among patients with preoperative Frankel deficit, improvement by postoperative day 10–14 was least frequent in the both-abnormalities group. This does not prove that posterior paraspinal degeneration causes impaired neurological improvement. Rather, the same systemic and axial condition reflected by the imaging phenotype may influence early mobilization and functional gain after decompression and stabilization. The endpoint was deliberately limited to postoperative day 10–14 and should not be interpreted as durable neurological recovery; later gains with rehabilitation or radiotherapy and subsequent deterioration from complications or disease progression were not captured.

Tumor biology remains central in MESCC prognosis. Hematologic/myeloma/lymphoma tumors were most frequent in the neither-abnormality group, whereas lung cancer and rapid-growth tumors were more frequent in the abnormal phenotype groups. This imbalance could have partly explained the crude survival gradient if not addressed. The persistence of the Goutallier association after Katagiri-style adjustment and the persistence of the combined phenotype in the solid-tumor-only sensitivity analysis suggest that measured tumor-biology variables did not fully explain the observed associations. However, residual confounding by unmeasured systemic disease burden, molecular status, prior oncological therapy, treatment response, and access to postoperative treatment remains possible.

Postoperative oncological treatment is an important determinant of the trajectory after MESCC surgery. Radiotherapy remains central to local disease control, while systemic treatment may include cytotoxic chemotherapy, targeted therapy, endocrine therapy, hematologic regimens, or immunotherapy according to tumor biology and prior treatment. In this cohort, postoperative systemic therapy within 90 days was less frequent in the both-abnormalities group than in the neither-abnormality group, but treatment receipt is conditioned by survival to treatment, wound recovery, performance status, and oncological eligibility; it therefore cannot be interpreted as an ordinary baseline confounder. Emerging minimally invasive local-control strategies such as spinal laser interstitial thermal therapy have shown promising reduction of epidural tumor burden in selected patients with compressive spinal metastases, although the available evidence remains based largely on observational series and requires further comparative validation [32].

These findings complement existing metastatic spine frameworks rather than replacing them. NOMS structures treatment selection around neurologic, oncologic, mechanical, and systemic considerations [2]. SINS and Bilsky grading characterize mechanical instability and epidural compression [3,4]. Katagiri, Tokuhashi, Tomita, modified Bauer, and NESMS integrate tumor biology and systemic condition into survival estimation [5,6,7,8,9]. A recent AO Spine critical appraisal concluded that existing frailty and sarcopenia tools in spinal oncology have poor or uncertain clinimetric properties [25]. The present findings support the same direction: routine-imaging muscle assessment is promising, but the field should move toward externally validated, multidimensional, outcome-specific tools rather than isolated single-muscle thresholds.

Several limitations should be acknowledged. First, this was a retrospective single-center study with a modest sample size. Second, image-derived measurements were not available in all patients, and technical non-measurability may introduce selection bias. Measurable and non-measurable patients did not differ significantly across the measured baseline domains summarized in Supplementary Table S2A,B, but these comparisons cannot exclude selection related to unmeasured disease burden or imaging availability. Third, posterior paraspinal Goutallier grading was recorded as the worst fatty infiltration grade of the combined multifidus/erector spinae compartment per side; separate muscle-specific grades were not available. Fourth, axial T2-weighted MRI provided a pragmatic semiquantitative grade rather than quantitative fat fraction; excellent inter-rater reliability does not substitute for external or criterion validation against Dixon, chemical-shift, quantitative fat-fraction MRI, or histology. Fifth, L3 is a systemic axial imaging level rather than the operated segment and may be influenced by age, degeneration, pain, disuse, or stenosis. Sixth, the lower-PVR stratum was defined using sex-specific cohort medians, and the both-abnormalities group contained only 21 patients; the large binary-outcome effect estimates and wide confidence intervals are therefore exploratory. Seventh, Karnofsky Performance Status and postoperative ECOG were not routinely documented and could not be reconstructed reliably; baseline ECOG was complete and was included in the principal adjusted models. Eighth, postoperative radiotherapy and systemic therapy are vulnerable to immortal-time and treatment-selection biases and were therefore described rather than entered as baseline adjustment covariates. Ninth, complication timing, discharge destination, and some detailed postoperative functional outcomes were not uniformly available; adjudicated complication and reoperation data were therefore summarized descriptively. Tenth, Frankel change at postoperative day 10–14 measures early neurological improvement rather than durable recovery. Finally, the findings lack external validation and should not be used as stand-alone criteria for surgical selection or operative strategy.

Despite these limitations, the study has several strengths: a consecutive surgical MESCC cohort, exclusion of intradural metastases, complete baseline ECOG data, explicit imaging timing criteria, paired assessment of psoas quantity and posterior paraspinal quality, use of routine imaging, explicit handling of technically non-measurable L3 anatomy, direct comparison of measurable and non-measurable patients, tumor-biology adjustment, solid-tumor-only sensitivity analysis, blinded reliability testing, adjudicated postoperative complication and reoperation data, and inclusion of early neurological improvement as a clinically relevant postoperative endpoint.

## 5. Conclusions

In patients undergoing surgery for MESCC, high-grade posterior paraspinal fatty infiltration was associated with inferior overall survival after adjustment for clinical status and tumor biology and with lower early neurological improvement. Psoas quantity alone separated unadjusted survival but did not show an adjusted association with survival in this cohort. An exploratory combined phenotype integrating the lower-PVR stratum with Goutallier grade 3–4 identified a small subgroup with poor observed outcomes, but its large and imprecise binary-outcome estimates require cautious interpretation. Posterior paraspinal Goutallier grading may serve as a pragmatic routine-imaging axial muscle-quality marker, but both the primary marker and the combined phenotype require independent external validation before clinical application and should not be used as stand-alone criteria for surgical selection or operative strategy.

## Figures and Tables

**Table 1:** Baseline clinical, laboratory, tumor, and operative characteristics according to posterior paraspinal Goutallier grade in patients with MESCC.

Variable	Overall (*n* = 112)	Goutallier 0–2 (*n* = 74)	Goutallier 3–4 (*n* = 38)	*p* Value
Age, years	62.0 [54.0, 70.0]	61.0 [54.0, 68.0]	68.5 [58.2, 74.0]	0.005
Male sex	74 (66.1)	48 (64.9)	26 (68.4)	0.707
ECOG performance status, median [IQR]	2 [1, 3]	2 [1, 3]	2 [2, 4]	0.009
ECOG 0	2 (1.8)	2 (2.7)	0 (0.0)	
ECOG 1	43 (38.4)	34 (45.9)	9 (23.7)	
ECOG 2	25 (22.3)	14 (18.9)	11 (28.9)	
ECOG 3	20 (17.9)	14 (18.9)	6 (15.8)	
ECOG 4	22 (19.6)	10 (13.5)	12 (31.6)	
Albumin, g/dL	3.70 [3.10, 4.10]	3.85 [3.50, 4.10]	3.35 [2.90, 3.88]	0.006
Prognostic nutritional index	37.5 [31.6, 41.5]	39.3 [35.4, 42.6]	33.8 [29.4, 38.7]	0.001
Neutrophil-to-lymphocyte ratio	4.02 [2.41, 5.81]	3.62 [2.27, 5.03]	4.47 [3.10, 8.18]	0.036
Primary tumor group				0.663
Hematologic/myeloma/lymphoma	27 (24.1)	20 (27.0)	7 (18.4)	
Favorable solid tumor	20 (17.9)	14 (18.9)	6 (15.8)	
Lung cancer	35 (31.2)	21 (28.4)	14 (36.8)	
Other solid tumor	30 (26.8)	19 (25.7)	11 (28.9)	
Katagiri-style tumor-growth group				0.492
Slow	47 (42.0)	34 (45.9)	13 (34.2)	
Moderate	13 (11.6)	8 (10.8)	5 (13.2)	
Rapid	52 (46.4)	32 (43.2)	20 (52.6)	
Bilsky grade 3 compression	54 (48.2)	33 (44.6)	21 (55.3)	0.285
Preoperative Frankel deficit	66 (58.9)	39 (52.7)	27 (71.1)	0.062
Preoperative ambulatory status (Frankel D/E)	78 (69.6)	54 (73.0)	24 (63.2)	0.285
SINS total	10.0 [8.0, 12.0]	9.0 [7.0, 12.0]	11.0 [8.2, 13.8]	0.052
Surgical strategy				0.226
Decompression only or decompression + augmentation	35 (31.2)	26 (35.1)	9 (23.7)	
Decompression + short instrumentation	26 (23.2)	16 (21.6)	10 (26.3)	
Decompression + long instrumentation	33 (29.5)	18 (24.3)	15 (39.5)	
Corpectomy or 360-degree reconstruction	18 (16.1)	14 (18.9)	4 (10.5)	
Instrumentation	77 (68.8)	48 (64.9)	29 (76.3)	0.216
Operative time, min	230 [158, 310]	235 [150, 332]	230 [172, 284]	0.597
Any transfusion	65 (58.0)	36 (48.6)	29 (76.3)	0.005

Values are presented as median [interquartile range] or *n* (%). Posterior paraspinal Goutallier grade refers to the patient-level maximum grade at L3, derived from the right and left multifidus/erector spinae compartments. ECOG is shown both as median [interquartile range] and as the full categorical distribution. *p* values are descriptive and were not used for variable selection. ECOG, Eastern Cooperative Oncology Group; SINS, Spinal Instability Neoplastic Score.

**Table 2:** Imaging body-composition measurement availability, exploratory phenotype construction, and posterior paraspinal Goutallier reliability.

Item	Value
Total MESCC cohort	133
Psoas-to-vertebral-body ratio available	119/133 (89.5)
Psoas muscle index available	119/133 (89.5)
Psoas radiodensity available	119/133 (89.5)
Posterior paraspinal Goutallier available	112/133 (84.2)
Complete exploratory combined axial muscle phenotype available	109/133 (82.0)
Goutallier grade distribution among measurable patients	
Grade 0	6/112 (5.4)
Grade 1	24/112 (21.4)
Grade 2	44/112 (39.3)
Grade 3	30/112 (26.8)
Grade 4	8/112 (7.1)
Goutallier 0–2	74/112 (66.1)
Goutallier 3–4	38/112 (33.9)
Lower-PVR stratum by sex-specific cohort median	59/119 (49.6)
Exploratory combined axial muscle phenotype among complete cases	
Neither abnormality	39/109 (35.8)
One abnormality	49/109 (45.0)
Both abnormalities	21/109 (19.3)
Inter-rater reliability, *n* = 40	
Right-sided grade: exact agreement	82.5%
Right-sided grade: within-one-grade agreement	100.0%
Right-sided grade: quadratic weighted kappa	0.907
Left-sided grade: exact agreement	77.5%
Left-sided grade: within-one-grade agreement	100.0%
Left-sided grade: quadratic weighted kappa	0.874
Patient-level maximum grade: exact agreement	80.0%
Patient-level maximum grade: within-one-grade agreement	100.0%
Patient-level maximum grade: quadratic weighted kappa	0.887

PVR was calculated as total bilateral psoas cross-sectional area divided by L3 vertebral body cross-sectional area. PMI was calculated as total bilateral psoas cross-sectional area divided by height squared and expressed as cm^2^/m^2^. Psoas radiodensity was recorded as mean Hounsfield units. The exploratory combined axial muscle phenotype was defined among patients with both PVR and posterior paraspinal Goutallier data. The lower-PVR stratum was defined using sex-specific cohort medians. Weighted kappa values are quadratic weighted Cohen kappa. PMI, psoas muscle index; PVR, psoas-to-vertebral-body ratio.

**Table 3:** Tumor biology, performance status, surgical strategy, postoperative treatment, and outcomes according to the exploratory combined axial muscle phenotype.

Variable	Neither Abnormality (*n* = 39)	One Abnormality (*n* = 49)	Both Abnormalities (*n* = 21)	*p* Value
Primary tumor group				0.129
Hematologic/myeloma/lymphoma	15 (38.5)	8 (16.3)	3 (14.3)	
Favorable solid tumor	7 (17.9)	9 (18.4)	3 (14.3)	
Lung cancer	7 (17.9)	20 (40.8)	8 (38.1)	
Other solid tumor	10 (25.6)	12 (24.5)	7 (33.3)	
Katagiri-style tumor-growth group				0.113
Slow	22 (56.4)	17 (34.7)	6 (28.6)	
Moderate	5 (12.8)	5 (10.2)	2 (9.5)	
Rapid	12 (30.8)	27 (55.1)	13 (61.9)	
ECOG performance status				0.007
ECOG 0	2 (5.1)	0 (0.0)	0 (0.0)	
ECOG 1	20 (51.3)	18 (36.7)	5 (23.8)	
ECOG 2	7 (17.9)	11 (22.4)	5 (23.8)	
ECOG 3	7 (17.9)	11 (22.4)	2 (9.5)	
ECOG 4	3 (7.7)	9 (18.4)	9 (42.9)	
Bilsky grade 3 compression	15 (38.5)	26 (53.1)	11 (52.4)	0.353
Preoperative Frankel deficit	24 (61.5)	24 (49.0)	15 (71.4)	0.184
Surgical strategy				0.648
Decompression only or decompression + augmentation	14 (35.9)	13 (26.5)	6 (28.6)	
Decompression + short instrumentation	9 (23.1)	12 (24.5)	4 (19.0)	
Decompression + long instrumentation	11 (28.2)	13 (26.5)	9 (42.9)	
Corpectomy or 360-degree reconstruction	5 (12.8)	11 (22.4)	2 (9.5)	
Instrumentation	25 (64.1)	36 (73.5)	15 (71.4)	0.626
Operative time, min	250 [180, 318]	230 [150, 305]	230 [170, 300]	0.641
Any transfusion	14 (35.9)	32 (65.3)	17 (81.0)	0.001
Postoperative length of stay, days	4 [3, 6]	5 [3, 8]	7 [4, 15]	NT
Any recorded perioperative/postoperative complication	13 (33.3)	15 (30.6)	10 (47.6)	NT
Wound-related complication	3 (7.7)	4 (8.2)	1 (4.8)	NT
Infectious complication	1 (2.6)	3 (6.1)	4 (19.0)	NT
Venous thromboembolism	2 (5.1)	0 (0.0)	2 (9.5)	NT
Early unplanned index-related reoperation	5 (12.8)	8 (16.3)	4 (19.0)	NT
Postoperative radiotherapy within 90 days	10 (25.6)	18 (36.7)	3 (14.3)	NT
Postoperative systemic therapy within 90 days	27 (69.2)	26 (53.1)	5 (23.8)	NT
In-hospital mortality	0 (0.0)	1 (2.0)	1 (4.8)	NT
Deaths during follow-up	18 (46.2)	34 (69.4)	17 (81.0)	0.014
90-day mortality	1 (2.6)	6 (12.2)	11 (52.4)	<0.001
Observed death within 365 days	15 (38.5)	26 (53.1)	16 (76.2)	0.020
KM-estimated 365-day survival	59.6% (45.6–78.0)	45.1% (32.8–62.1)	23.8% (11.1–51.2)	
Median survival, days (95% CI)	658 (251-NR)	306 (190–578)	73 (57-NR)	<0.001
Restricted mean survival time to 365 days	289.4	250.0	148.2	
Postoperative ambulatory status (Frankel D/E), POD10-14	36 (92.3)	37 (75.5)	13 (61.9)	NT
Ambulatory conversion among preoperatively nonambulatory patients	6/9 (66.7)	6/17 (35.3)	0/7 (0.0)	NT
Early Frankel improvement among patients with deficit, POD10-14	17/24 (70.8)	12/24 (50.0)	2/15 (13.3)	NT

The exploratory combined axial muscle phenotype was defined as neither abnormality, one abnormality, or both the lower-PVR stratum and Goutallier grade 3–4. The lower-PVR stratum was defined using sex-specific cohort medians. Recorded perioperative/postoperative complications and reoperations were manually adjudicated from the available structured and free-text fields; planned staged procedures, operations for a new metastatic level, and clearly preoperative events were excluded from index-related early unplanned reoperation counts. Wound-related complications included wound drainage, infection/abscess, or wound revision; infectious complications included documented postoperative wound infection/abscess, pneumonia, sepsis, or urinary infection; venous thromboembolism included postoperative DVT or thrombophlebitis. Postoperative radiotherapy, systemic therapy, adjudicated complications, and denominator-dependent functional outcomes were not formally tested because of survival-to-treatment, follow-up, timing, and denominator considerations. CI, confidence interval; KM, Kaplan–Meier; NR, not reached; PVR, psoas-to-vertebral-body ratio; RMST, restricted mean survival time; VTE, venous thromboembolism; NT, not tested.

**Table 4:** Main and sensitivity models for survival, 90-day mortality, and early neurological improvement.

Outcome/Model	Exposure Contrast	*n*/Events	Effect Estimate (95% CI)	*p* Value
Overall survival: primary clinical/Katagiri Cox model	Goutallier 3–4 vs. 0–2	112/72 deaths	HR 1.80 (1.06–3.07)	0.029
Overall survival: PNI/log-NLR Cox model	Goutallier 3–4 vs. 0–2	112/72 deaths	HR 1.87 (1.10–3.19)	0.021
Overall survival: four-group tumor Cox model	Goutallier 3–4 vs. 0–2	112/72 deaths	HR 1.90 (1.11–3.24)	0.019
Overall survival: surgical-strategy sensitivity Cox model	Goutallier 3–4 vs. 0–2	112/72 deaths	HR 2.01 (1.15–3.51)	0.014
Overall survival: ordinal Goutallier Cox model	Per one-grade increase	112/72 deaths	HR 1.43 (1.09–1.87)	0.010
Overall survival: primary clinical/Katagiri Cox model	Lower-PVR vs. higher-PVR stratum	119/75 deaths	HR 1.24 (0.73–2.10)	0.424
Overall survival: primary clinical/Katagiri Cox model	Low vs. higher PMI	119/75 deaths	HR 1.28 (0.76–2.15)	0.347
Overall survival: primary clinical/Katagiri Cox model	One abnormality vs. neither	109/69 deaths	HR 1.23 (0.60–2.55)	0.574
Overall survival: primary clinical/Katagiri Cox model	Both abnormalities vs. neither	109/69 deaths	HR 2.64 (1.15–6.03)	0.021
Overall survival: PNI/log-NLR Cox model	Both abnormalities vs. neither	109/69 deaths	HR 3.15 (1.33–7.44)	0.009
Overall survival: surgical-strategy sensitivity Cox model	Both abnormalities vs. neither	109/69 deaths	HR 3.35 (1.39–8.07)	0.007
Overall survival: solid-tumor-only Cox model	Goutallier 3–4 vs. 0–2	85/63 deaths	HR 1.71 (0.96–3.06)	0.069
Overall survival: solid-tumor-only Cox model	Both abnormalities vs. neither	83/61 deaths	HR 2.71 (1.07–6.85)	0.035
90-day mortality: adjusted Firth logistic model	Goutallier 3–4 vs. 0–2	112/18 deaths	OR 4.16 (1.29–15.16)	0.017
90-day mortality: adjusted Firth logistic model	Both abnormalities vs. neither	109/18 deaths	OR 19.26 (2.69–274.47)	0.002
Early Frankel improvement: adjusted Firth logistic model	Goutallier 3–4 vs. 0–2	66/32 improvements	OR 0.28 (0.08–0.83)	0.022
Early Frankel improvement: adjusted Firth logistic model	Both abnormalities vs. neither	63/31 improvements	OR 0.14 (0.02–0.68)	0.014

Cox proportional hazards models report hazard ratios for overall survival. Firth bias-reduced logistic regression models report odds ratios for 90-day mortality and early Frankel improvement. The primary Cox model adjusted for age, sex, ECOG performance status, albumin, Bilsky grade 3 compression, and Katagiri-style tumor-growth group. ECOG was modeled per one-grade increase. The PNI/log-NLR model replaced albumin with PNI and added log-transformed NLR. PNI was modeled per five-point decrease. The surgical-strategy sensitivity model additionally included broad operative strategy. Firth models for 90-day mortality adjusted for age, sex, ECOG performance status, albumin, Bilsky grade 3 compression, and Katagiri-style tumor-growth group. Firth models for early Frankel improvement adjusted for age, sex, ECOG performance status, Bilsky grade 3 compression, Katagiri-style tumor-growth group, and preoperative Frankel grade. Frankel-improvement analyses included only patients with a preoperative Frankel deficit. The combined-phenotype binary-outcome models are exploratory because of the small both-abnormalities subgroup and wide confidence intervals. CI, confidence interval; ECOG, Eastern Cooperative Oncology Group; HR, hazard ratio; NLR, neutrophil-to-lymphocyte ratio; OR, odds ratio; PMI, psoas muscle index; PNI, prognostic nutritional index; PVR, psoas-to-vertebral-body ratio.

## Data Availability

The data analyzed during the current study are available from the corresponding author on reasonable request, subject to institutional and ethical restrictions. The dataset cannot be publicly deposited because of patient privacy and institutional data-sharing restrictions.

## Supplementary Materials

The following supporting information can be downloaded at https://www.mdpi.com/article/10.3390/medicina62091720/s1, Supplementary Table S1. Primary pathology tumor-biology mapping, Supplementary Table S2A. Baseline comparison according to posterior paraspinal Goutallier measurability, Supplementary Table S2B. Baseline comparison according to complete axial body-composition phenotype availability, Supplementary Table S3A. Kaplan–Meier 365-day fixed-time survival estimates, Supplementary Table S3B. Restricted mean survival time to 365 days, Supplementary Table S4. Continuous sex-standardized psoas Cox sensitivity models, Supplementary Table S5. Proportional-hazards assumption tests, Supplementary Table S6. Full Cox proportional hazards model coefficients, Supplementary Table S7. Full Firth logistic regression model coefficients.

## References

[1] Patchell R.A., Tibbs P.A., Regine W.F., Payne R., Saris S., Kryscio R.J., Mohiuddin M., Young B. (2005). Direct Decompressive Surgical Resection in the Treatment of Spinal Cord Compression Caused by Metastatic Cancer: A Randomised Trial. Lancet.

[2] Laufer I., Rubin D.G., Lis E., Cox B.W., Stubblefield M.D., Yamada Y., Bilsky M.H. (2013). The NOMS Framework: Approach to the Treatment of Spinal Metastatic Tumors. Oncologist.

[3] Bilsky M.H., Laufer I., Fourney D.R., Groff M., Schmidt M.H., Varga P.P., Vrionis F.D., Yamada Y., Gerszten P.C., Kuklo T.R. (2010). Reliability Analysis of the Epidural Spinal Cord Compression Scale. J. Neurosurg. Spine.

[4] Fisher C.G., DiPaola C.P., Ryken T.C., Bilsky M.H., Shaffrey C.I., Berven S.H., Harrop J.S., Fehlings M.G., Boriani S., Chou D. (2010). A Novel Classification System for Spinal Instability in Neoplastic Disease: An Evidence-Based Approach and Expert Consensus from the Spine Oncology Study Group. Spine.

[5] Tomita K., Kawahara N., Kobayashi T., Yoshida A., Murakami H., Akamaru T. (2001). Surgical Strategy for Spinal Metastases. Spine.

[6] Tokuhashi Y., Matsuzaki H., Oda H., Oshima M., Ryu J. (2005). A Revised Scoring System for Preoperative Evaluation of Metastatic Spine Tumor Prognosis. Spine.

[7] Bauer H.C., Wedin R. (1995). Survival after Surgery for Spinal and Extremity Metastases. Prognostication in 241 Patients. Acta Orthop. Scand..

[8] Katagiri H., Okada R., Takagi T., Takahashi M., Murata H., Harada H., Nishimura T., Asakura H., Ogawa H. (2014). New Prognostic Factors and Scoring System for Patients with Skeletal Metastasis. Cancer Med..

[9] Schoenfeld A.J., Ferrone M.L., Schwab J.H., Blucher J.A., Barton L.B., Tobert D.G., Chi J.H., Shin J.H., Kang J.D., Harris M.B. (2021). Prospective Validation of a Clinical Prediction Score for Survival in Patients with Spinal Metastases: The New England Spinal Metastasis Score. Spine J. Off. J. N. Am. Spine Soc..

[10] Prado C.M.M., Lieffers J.R., McCargar L.J., Reiman T., Sawyer M.B., Martin L., Baracos V.E. (2008). Prevalence and Clinical Implications of Sarcopenic Obesity in Patients with Solid Tumours of the Respiratory and Gastrointestinal Tracts: A Population-Based Study. Lancet Oncol..

[11] Mourtzakis M., Prado C.M.M., Lieffers J.R., Reiman T., McCargar L.J., Baracos V.E. (2008). A Practical and Precise Approach to Quantification of Body Composition in Cancer Patients Using Computed Tomography Images Acquired during Routine Care. Appl. Physiol. Nutr. Metab. Physiol. Appl. Nutr. Metab..

[12] Martin L., Birdsell L., Macdonald N., Reiman T., Clandinin M.T., McCargar L.J., Murphy R., Ghosh S., Sawyer M.B., Baracos V.E. (2013). Cancer Cachexia in the Age of Obesity: Skeletal Muscle Depletion Is a Powerful Prognostic Factor, Independent of Body Mass Index. J. Clin. Oncol. Off. J. Am. Soc. Clin. Oncol..

[13] Shachar S.S., Williams G.R., Muss H.B., Nishijima T.F. (2016). Prognostic Value of Sarcopenia in Adults with Solid Tumours: A Meta-Analysis and Systematic Review. Eur. J. Cancer.

[14] Caan B.J., Cespedes Feliciano E.M., Prado C.M., Alexeeff S., Kroenke C.H., Bradshaw P., Quesenberry C.P., Weltzien E.K., Castillo A.L., Olobatuyi T.A. (2018). Association of Muscle and Adiposity Measured by Computed Tomography With Survival in Patients With Nonmetastatic Breast Cancer. JAMA Oncol..

[15] Bourassa-Moreau É., Versteeg A., Moskven E., Charest-Morin R., Flexman A., Ailon T., Dalkilic T., Fisher C., Dea N., Boyd M. (2020). Sarcopenia, but Not Frailty, Predicts Early Mortality and Adverse Events after Emergent Surgery for Metastatic Disease of the Spine. Spine J. Off. J. N. Am. Spine Soc..

[16] Zakaria H.M., Wilkinson B.M., Pennington Z., Saadeh Y.S., Lau D., Chandra A., Ahmed A.K., Macki M., Anand S.K., Abouelleil M.A. (2020). Sarcopenia as a Prognostic Factor for 90-Day and Overall Mortality in Patients Undergoing Spine Surgery for Metastatic Tumors: A Multicenter Retrospective Cohort Study. Neurosurgery.

[17] Hu M.-H., Yen H.-K., Chen I.-H., Wu C.-H., Chen C.-W., Yang J.-J., Wang Z.-Y., Yen M.-H., Yang S.-H., Lin W.-H. (2022). Decreased Psoas Muscle Area Is a Prognosticator for 90-Day and 1-Year Survival in Patients Undergoing Surgical Treatment for Spinal Metastasis. Clin. Nutr..

[18] Bongers M.E.R., Groot O.Q., Buckless C.G., Kapoor N.D., Twining P.K., Schwab J.H., Torriani M., Bredella M.A. (2022). Body Composition Predictors of Mortality on Computed Tomography in Patients with Spinal Metastases Undergoing Surgical Treatment. Spine J. Off. J. N. Am. Spine Soc..

[19] Pigneur F., Di Palma M., Raynard B., Guibal A., Cohen F., Daidj N., Aziza R., El Hajjam M., Louis G., Goldwasser F. (2023). Psoas Muscle Index Is Not Representative of Skeletal Muscle Index for Evaluating Cancer Sarcopenia. J. Cachexia Sarcopenia Muscle.

[20] He K., Head J., Mouchtouris N., Hines K., Shea P., Schmidt R., Hoelscher C., Stricsek G., Harrop J., Sharan A. (2020). The Implications of Paraspinal Muscle Atrophy in Low Back Pain, Thoracolumbar Pathology, and Clinical Outcomes After Spine Surgery: A Review of the Literature. Glob. Spine J..

[21] Pezolato A., de Vasconcelos E.E., Defino H.L.A., Nogueira-Barbosa M.H. (2012). Fat Infiltration in the Lumbar Multifidus and Erector Spinae Muscles in Subjects with Sway-Back Posture. Eur. Spine J. Off. Publ. Eur. Spine Soc. Eur. Spinal Deform. Soc. Eur. Sect. Cerv. Spine Res. Soc..

[22] Goutallier D., Postel J.M., Bernageau J., Lavau L., Voisin M.C. (1994). Fatty Muscle Degeneration in Cuff Ruptures. Pre- and Postoperative Evaluation by CT Scan. Clin. Orthop..

[23] Battaglia P.J., Maeda Y., Welk A., Hough B., Kettner N. (2014). Reliability of the Goutallier Classification in Quantifying Muscle Fatty Degeneration in the Lumbar Multifidus Using Magnetic Resonance Imaging. J. Manip. Physiol. Ther..

[24] Mandelli F., Nüesch C., Zhang Y., Halbeisen F., Schären S., Mündermann A., Netzer C. (2021). Assessing Fatty Infiltration of Paraspinal Muscles in Patients With Lumbar Spinal Stenosis: Goutallier Classification and Quantitative MRI Measurements. Front. Neurol..

[25] MacLean M.A., Charles A.J., Georgiopoulos M., Phinney J., Charest-Morin R., Goodwin R., Laufer I., Fehlings M.G., Shin J., Dea N. (2025). A Critical Appraisal of the Application of Frailty and Sarcopenia in the Spinal Oncology Population. Glob. Spine J..

[26] Costanzo R., Brunasso L., Paolini F., Benigno U.E., Porzio M., Giammalva G.R., Gerardi R.M., Umana G.E., di Bonaventura R., Sturiale C.L. (2022). Spinal Tractography as a Potential Prognostic Tool in Spinal Cord Injury: A Systematic Review. World Neurosurg..

[27] Kubota H., Soejima T., Sulaiman N.S., Sekii S., Matsumoto Y., Ota Y., Tsujino K., Fujita I., Fujimoto T., Morishita M. (2019). Predicting the Survival of Patients with Bone Metastases Treated with Radiation Therapy: A Validation Study of the Katagiri Scoring System. Radiat. Oncol..

[28] Royston P., Parmar M.K.B. (2013). Restricted Mean Survival Time: An Alternative to the Hazard Ratio for the Design and Analysis of Randomized Trials with a Time-to-Event Outcome. BMC Med. Res. Methodol..

[29] Pielkenrood B.J., van Urk P.R., van der Velden J.M., Kasperts N., Verhoeff J.J.C., Bol G.H., Verkooijen H.M., Verlaan J.J. (2020). Impact of Body Fat Distribution and Sarcopenia on the Overall Survival in Patients with Spinal Metastases Receiving Radiotherapy Treatment: A Prospective Cohort Study. Acta Oncol..

[30] Lee C.-C., Tseng T.-E., Chang R.-F., Yen H.-K., Chen Y.-A., Chen Y.-Y., Wu C.-H., Hu M.-H., Yen M.-H., Bongers M. (2024). Psoas Muscle Area Is an Independent Survival Prognosticator in Patients Undergoing Surgery for Long-Bone Metastases. Cancer Med..

[31] Kulkarni R., Walton C., D’Amico C., Bertolino M., Silvestre J., Rivas G., Lewis S., Nielsen C., Glaser J., Reitman C. (2026). Extent of Fatty Infiltration of Lumbar Paraspinal Muscles as a Proxy for Frailty and Its Relationship with Perioperative Outcomes in Patients Undergoing Elective Spinal Surgery. Glob. Spine J..

[32] Cardia A., Cannizzaro D., Stefini R., Chibbaro S., Ganau M., Zaed I. (2023). The Efficacy of Laser Interstitial Thermal Therapy in the Management of Spinal Metastases: A Systematic Review of the Literature. Neurol. Sci..

