# Supplementary material for: Preoperative Posterior Paraspinal Fatty Infiltration and an Exploratory Axial Muscle Imaging Phenotype in Surgically Treated Metastatic Epidural Spinal Cord Compression: A Retrospective Cohort Study
Source: Medicina (Kaunas). 2026 Sep 7;62(9):1720. doi: 10.3390/medicina62091720 (PMC13609030; doi:10.3390/medicina62091720)
Supplement: Supplementary file 1 [file medicina-62-01720-s001.zip › medicina-4430116-supplementary.pdf]

Supplementary Tables

Supplementary Table S1. Primary pathology tumor-biology mapping.

Primary pathology	n	Four-group classification	Katagiri-style growth group
Multiple myeloma	21	Hematologic / myeloma / lymphoma	Slow
Lung adenocarcinoma	20	Lung cancer	Rapid
Breast carcinoma	10	Favorable solid tumor	Slow
Prostate adenocarcinoma	10	Favorable solid tumor	Slow
Small-cell lung cancer	9	Lung cancer	Rapid
Lymphoma	9	Hematologic / myeloma / lymphoma	Slow
Colorectal adenocarcinoma	6	Other solid tumor	Rapid
Pancreatic carcinoma	6	Other solid tumor	Rapid
Lung carcinoma, not otherwise specified	4	Lung cancer	Rapid
Clear-cell renal cell carcinoma	4	Other solid tumor	Moderate
Renal cell carcinoma, not otherwise specified	4	Other solid tumor	Moderate
Differentiated thyroid carcinoma	4	Favorable solid tumor	Slow
Lung squamous cell carcinoma	3	Lung cancer	Rapid
Gastric carcinoma	3	Other solid tumor	Rapid
Lung adenosquamous carcinoma	2	Lung cancer	Rapid
Laryngeal / head-and-neck carcinoma	2	Other solid tumor	Rapid
Pancreatobiliary carcinoma	2	Other solid tumor	Rapid
Uterine / gynecologic carcinoma	2	Other solid tumor	Moderate
Hepatocellular / hepatobiliary carcinoma	2	Other solid tumor	Rapid
Testicular / germ-cell tumor	2	Favorable solid tumor	Slow
Adrenal carcinoma	1	Other solid tumor	Rapid
Combined neuroendocrine carcinoma of lung	1	Lung cancer	Rapid
Non-small-cell lung cancer, not otherwise specified	1	Lung cancer	Rapid
Liposarcoma	1	Other solid tumor	Moderate
Malignant melanoma	1	Other solid tumor	Rapid
Poorly differentiated neuroendocrine carcinoma	1	Other solid tumor	Rapid
Sarcoma, not otherwise specified	1	Other solid tumor	Moderate
Mixed anaplastic/papillary/follicular thyroid carcinoma	1	Other solid tumor	Rapid

Counts reflect the full extradural spinal metastasis cohort (n = 133). Formal pathology labels were harmonized from institutional pathology descriptors for tumor-biology grouping.

Supplementary Table S2A. Baseline comparison according to posterior paraspinal Goutallier measurability.

Variable	Goutallier not measurable (n=21)	Goutallier measurable (n=112)	p value
n	21	112	
Age, years	60.0 [47.0, 67.0]	62.0 [54.0, 70.0]	0.230
ECOG performance status	2 [2, 3]	2 [1, 3]	0.601
Albumin, g/dL	3.50 [3.40, 3.90]	3.70 [3.10, 4.10]	0.704
Overall survival, days	137 [69, 434]	230 [120, 472]	0.300
Male sex	18 (85.7)	74 (66.1)	0.074
Bilsky grade 3 compression	8 (38.1)	54 (48.2)	0.394
Preoperative Frankel deficit	14 (66.7)	66 (58.9)	0.506
Primary tumor group			0.495
Hematologic / myeloma / lymphoma	3 (14.3)	27 (24.1)	
Favorable solid tumor	6 (28.6)	20 (17.9)	
Lung cancer	5 (23.8)	35 (31.2)	
Other solid tumor	7 (33.3)	30 (26.8)	
Katagiri-style tumor-growth group			0.630
Slow	9 (42.9)	47 (42.0)	
Moderate	1 (4.8)	13 (11.6)	
Rapid	11 (52.4)	52 (46.4)	

Values are median [IQR] or n (%). P values are descriptive.

Supplementary Table S2B. Baseline comparison according to complete axial body-composition phenotype availability.

Variable	Incomplete phenotype (n=24)	Complete phenotype (n=109)	p value
n	24	109	
Age, years	61.5 [47.8, 68.2]	62.0 [54.0, 70.0]	0.370
ECOG performance status	2 [2, 3]	2 [1, 3]	0.406
Albumin, g/dL	3.50 [3.27, 3.92]	3.70 [3.10, 4.10]	0.594
Overall survival, days	186 [73, 362]	227 [120, 473]	0.282
Male sex	20 (83.3)	72 (66.1)	0.097
Bilsky grade 3 compression	10 (41.7)	52 (47.7)	0.591
Preoperative Frankel deficit	17 (70.8)	63 (57.8)	0.238
Primary tumor group			0.394
Hematologic / myeloma / lymphoma	4 (16.7)	26 (23.9)	
Favorable solid tumor	7 (29.2)	19 (17.4)	
Lung cancer	5 (20.8)	35 (32.1)	
Other solid tumor	8 (33.3)	29 (26.6)	
Katagiri-style tumor-growth group			0.885
Slow	11 (45.8)	45 (41.3)	
Moderate	2 (8.3)	12 (11.0)	
Rapid	11 (45.8)	52 (47.7)	

Values are median [IQR] or n (%). P values are descriptive.

Supplementary Table S3A. Kaplan-Meier 365-day fixed-time survival estimates.

Analysis	Stratum	n at risk at 365 days	events by 365 days	365-day survival (95% CI)
Goutallier group	Goutallier 0-2	29	35	50.2% (39.6-63.6)
Goutallier group	Goutallier 3-4	10	25	33.5% (21.2-52.8)
Axial muscle-reserve phenotype	Neither abnormality	18	15	59.6% (45.6-78.0)
Axial muscle-reserve phenotype	One abnormality	18	26	45.1% (32.8-62.1)
Axial muscle-reserve phenotype	Both abnormalities	3	16	23.8% (11.1-51.2)
Low PVR group	Higher PVR	28	25	56.8% (45.3-71.3)
Low PVR group	Low PVR	15	37	35.5% (24.8-50.7)

Survival estimates are Kaplan-Meier estimates at 365 days.

Supplementary Table S3B. Restricted mean survival time to 365 days.

Comparison	RMST reference, days	RMST comparison, days	Difference, days (95% CI)	p value
Goutallier 3-4 vs Goutallier 0-2	268.0	195.9	-72.1 (-123.2 to -21.0)	0.006
Low PVR vs Higher PVR	278.7	206.5	-72.2 (-117.8 to -26.6)	0.002
One abnormality vs Neither abnormality	289.4	250.0	-39.4 (-87.4 to 8.6)	0.108
Both abnormalities vs Neither abnormality	289.4	148.2	-141.2 (-206.5 to -75.8)	<0.001

RMST differences are expressed as comparison group minus reference group.

Supplementary Table S4. Continuous sex-standardized psoas Cox sensitivity models.

Model	Exposure	n/events	HR (95% CI)	p value
Continuous sex-standardized PVR model	PVR, per sex-specific SD	119/75	1.04 (0.82-1.31)	0.751
Continuous sex-standardized PMI model	PMI, per sex-specific SD	119/75	0.98 (0.74-1.29)	0.877
Continuous sex-standardized psoas HU model	Psoas HU, per sex-specific SD	119/75	1.13 (0.87-1.45)	0.362

Each exposure was modeled per sex-specific standard deviation in a Cox model adjusted for age, sex, ECOG performance status, albumin, Bilsky grade 3 compression, and Katagiri-style tumor-growth group.

Supplementary Table S5. Proportional-hazards assumption tests.

Model	Term	chisq	p value
Goutallier 3-4: primary clinical/Katagiri model	Goutallier 3-4 vs 0-2	0.121	0.728
Goutallier 3-4: primary clinical/Katagiri model	Age, per year	0.685	0.408
Goutallier 3-4: primary clinical/Katagiri model	Sex	0.455	0.500
Goutallier 3-4: primary clinical/Katagiri model	ECOG performance status	0.214	0.643
Goutallier 3-4: primary clinical/Katagiri model	Albumin, g/dL	2.660	0.103
Goutallier 3-4: primary clinical/Katagiri model	Bilsky grade 3 compression	0.562	0.453
Goutallier 3-4: primary clinical/Katagiri model	Katagiri growth group	2.728	0.256
Goutallier 3-4: primary clinical/Katagiri model	GLOBAL	8.206	0.414
Goutallier 3-4: PNI/log-NLR model	Goutallier 3-4 vs 0-2	0.004	0.947
Goutallier 3-4: PNI/log-NLR model	Age, per year	0.941	0.332
Goutallier 3-4: PNI/log-NLR model	Sex	0.879	0.348
Goutallier 3-4: PNI/log-NLR model	ECOG performance status	0.212	0.645
Goutallier 3-4: PNI/log-NLR model	PNI	2.979	0.084
Goutallier 3-4: PNI/log-NLR model	Log-NLR	4.196	0.041
Goutallier 3-4: PNI/log-NLR model	Bilsky grade 3 compression	0.403	0.526
Goutallier 3-4: PNI/log-NLR model	Katagiri growth group	2.227	0.328
Goutallier 3-4: PNI/log-NLR model	GLOBAL	13.249	0.152
Goutallier 3-4: four-group tumor model	Goutallier 3-4 vs 0-2	0.116	0.734
Goutallier 3-4: four-group tumor model	Age, per year	1.001	0.317
Goutallier 3-4: four-group tumor model	Sex	1.083	0.298
Goutallier 3-4: four-group tumor model	ECOG performance status	0.110	0.740
Goutallier 3-4: four-group tumor model	Albumin, g/dL	2.096	0.148
Goutallier 3-4: four-group tumor model	Bilsky grade 3 compression	0.284	0.594
Goutallier 3-4: four-group tumor model	Primary tumor group	0.593	0.898
Goutallier 3-4: four-group tumor model	GLOBAL	7.525	0.583
Goutallier 3-4: surgical-strategy sensitivity model	Goutallier 3-4 vs 0-2	0.127	0.721
Goutallier 3-4: surgical-strategy sensitivity model	Age, per year	1.069	0.301
Goutallier 3-4: surgical-strategy sensitivity model	Sex	0.429	0.512
Goutallier 3-4: surgical-strategy sensitivity model	ECOG performance status	0.069	0.792
Goutallier 3-4: surgical-strategy sensitivity model	Albumin, g/dL	2.380	0.123
Goutallier 3-4: surgical-strategy sensitivity model	Bilsky grade 3 compression	0.362	0.547
Goutallier 3-4: surgical-strategy sensitivity model	Katagiri growth group	3.333	0.189
Goutallier 3-4: surgical-strategy sensitivity model	Surgical strategy	0.620	0.892
Goutallier 3-4: surgical-strategy sensitivity model	GLOBAL	10.057	0.525
Ordinal Goutallier per-grade model	Goutallier grade, per one-grade increase	0.322	0.570
Ordinal Goutallier per-grade model	Age, per year	0.684	0.408
Ordinal Goutallier per-grade model	Sex	0.423	0.516

Model	Term	chisq	p value
Ordinal Goutallier per-grade model	ECOG performance status	0.103	0.749
Ordinal Goutallier per-grade model	Albumin, g/dL	2.671	0.102
Ordinal Goutallier per-grade model	Bilsky grade 3 compression	0.451	0.502
Ordinal Goutallier per-grade model	Katagiri growth group	2.846	0.241
Ordinal Goutallier per-grade model	GLOBAL	8.627	0.375
Axial phenotype primary model	axial muscle reserve phenotype	4.972	0.083
Axial phenotype primary model	Age, per year	0.652	0.419
Axial phenotype primary model	Sex	0.376	0.540
Axial phenotype primary model	ECOG performance status	0.324	0.569
Axial phenotype primary model	Albumin, g/dL	2.220	0.136
Axial phenotype primary model	Bilsky grade 3 compression	0.931	0.335
Axial phenotype primary model	Katagiri growth group	1.342	0.511
Axial phenotype primary model	GLOBAL	11.718	0.230
Axial phenotype PNI/log-NLR model	axial muscle reserve phenotype	4.007	0.135
Axial phenotype PNI/log-NLR model	Age, per year	0.990	0.320
Axial phenotype PNI/log-NLR model	Sex	0.660	0.416
Axial phenotype PNI/log-NLR model	ECOG performance status	0.312	0.576
Axial phenotype PNI/log-NLR model	PNI	2.819	0.093
Axial phenotype PNI/log-NLR model	Log-NLR	3.634	0.057
Axial phenotype PNI/log-NLR model	Bilsky grade 3 compression	0.721	0.396
Axial phenotype PNI/log-NLR model	Katagiri growth group	1.088	0.580
Axial phenotype PNI/log-NLR model	GLOBAL	13.104	0.218
Axial phenotype surgical-strategy sensitivity model	axial muscle reserve phenotype	4.352	0.113
Axial phenotype surgical-strategy sensitivity model	Age, per year	1.058	0.304
Axial phenotype surgical-strategy sensitivity model	Sex	0.246	0.620
Axial phenotype surgical-strategy sensitivity model	ECOG performance status	0.033	0.856
Axial phenotype surgical-strategy sensitivity model	Albumin, g/dL	1.717	0.190
Axial phenotype surgical-strategy sensitivity model	Bilsky grade 3 compression	0.640	0.424
Axial phenotype surgical-strategy sensitivity model	Katagiri growth group	1.397	0.497
Axial phenotype surgical-strategy sensitivity model	Surgical strategy	1.282	0.734
Axial phenotype surgical-strategy sensitivity model	GLOBAL	15.452	0.218

Schoenfeld residual-based tests are shown for each Cox model. Global p values are included where available.

Supplementary Table S6. Full Cox proportional hazards model coefficients.

Model	Term	n/events	HR (95% CI)	p value
Goutallier 3-4: primary clinical/Katagiri model	Goutallier 3-4 vs 0-2	112/72	1.80 (1.06-3.07)	0.029
Goutallier 3-4: primary clinical/Katagiri model	Age, per year	112/72	0.99 (0.96-1.01)	0.206
Goutallier 3-4: primary clinical/Katagiri model	Male sex	112/72	0.55 (0.31-0.95)	0.033
Goutallier 3-4: primary clinical/Katagiri model	ECOG performance status	112/72	1.58 (1.21-2.07)	<0.001
Goutallier 3-4: primary clinical/Katagiri model	Albumin, g/dL	112/72	0.84 (0.58-1.23)	0.381
Goutallier 3-4: primary clinical/Katagiri model	Bilsky grade 3 compression	112/72	1.45 (0.83-2.53)	0.197
Goutallier 3-4: primary clinical/Katagiri model	Katagiri moderate vs slow	112/72	4.02 (1.78-9.10)	<0.001
Goutallier 3-4: primary clinical/Katagiri model	Katagiri rapid vs slow	112/72	4.54 (2.48-8.32)	<0.001
Goutallier 3-4: PNI/log-NLR model	Goutallier 3-4 vs 0-2	112/72	1.87 (1.10-3.19)	0.021
Goutallier 3-4: PNI/log-NLR model	Age, per year	112/72	0.99 (0.96-1.01)	0.231
Goutallier 3-4: PNI/log-NLR model	Male sex	112/72	0.51 (0.30-0.88)	0.015
Goutallier 3-4: PNI/log-NLR model	ECOG performance status	112/72	1.55 (1.19-2.01)	0.001
Goutallier 3-4: PNI/log-NLR model	PNI	112/72	1.00 (1.00-1.00)	0.542
Goutallier 3-4: PNI/log-NLR model	Log-NLR	112/72	1.44 (1.03-2.03)	0.034
Goutallier 3-4: PNI/log-NLR model	Bilsky grade 3 compression	112/72	1.46 (0.84-2.53)	0.177
Goutallier 3-4: PNI/log-NLR model	Katagiri moderate vs slow	112/72	3.92 (1.70-9.03)	0.001
Goutallier 3-4: PNI/log-NLR model	Katagiri rapid vs slow	112/72	4.64 (2.49-8.64)	<0.001
Goutallier 3-4: four-group tumor model	Goutallier 3-4 vs 0-2	112/72	1.90 (1.11-3.24)	0.019
Goutallier 3-4: four-group tumor model	Age, per year	112/72	0.99 (0.97-1.01)	0.247
Goutallier 3-4: four-group tumor model	Male sex	112/72	0.63 (0.36-1.10)	0.101
Goutallier 3-4: four-group tumor model	ECOG performance status	112/72	1.64 (1.24-2.16)	<0.001
Goutallier 3-4: four-group tumor model	Albumin, g/dL	112/72	0.84 (0.57-1.24)	0.384
Goutallier 3-4: four-group tumor model	Bilsky grade 3 compression	112/72	1.42 (0.80-2.51)	0.228
Goutallier 3-4: four-group tumor model	Favorable solid tumor vs hematologic/myeloma/lymphoma	112/72	2.12 (0.83-5.40)	0.116
Goutallier 3-4: four-group tumor model	Lung cancer vs hematologic/myeloma/lymphoma	112/72	5.12 (2.31-11.35)	<0.001
Goutallier 3-4: four-group tumor model	Other solid tumor vs hematologic/myeloma/lymphoma	112/72	7.51 (3.31-17.07)	<0.001
Goutallier 3-4: surgical-strategy sensitivity model	Goutallier 3-4 vs 0-2	112/72	2.01 (1.15-3.51)	0.014
Goutallier 3-4: surgical-strategy sensitivity model	Age, per year	112/72	0.98 (0.96-1.01)	0.151
Goutallier 3-4: surgical-strategy sensitivity model	Male sex	112/72	0.56 (0.32-0.98)	0.041
Goutallier 3-4: surgical-strategy sensitivity model	ECOG performance status	112/72	1.62 (1.23-2.13)	<0.001
Goutallier 3-4: surgical-strategy sensitivity model	Albumin, g/dL	112/72	0.83 (0.57-1.21)	0.336
Goutallier 3-4: surgical-strategy sensitivity model	Bilsky grade 3 compression	112/72	1.74 (0.93-3.24)	0.082
Goutallier 3-4: surgical-strategy sensitivity model	Katagiri moderate vs slow	112/72	3.77 (1.60-8.90)	0.002
Goutallier 3-4: surgical-strategy sensitivity model	Katagiri rapid vs slow	112/72	4.68 (2.53-8.66)	<0.001
Goutallier 3-4: surgical-strategy sensitivity model	Decompression + short instrumentation vs decompression-only/augmentation	112/72	0.83 (0.42-1.62)	0.577

Model	Term	n/events	HR (95% CI)	p value
Goutallier 3-4: surgical-strategy sensitivity model	Decompression + long instrumentation vs decompression-only/augmentation	112/72	0.64 (0.32-1.30)	0.217
Goutallier 3-4: surgical-strategy sensitivity model	Corpectomy/360 reconstruction vs decompression-only/augmentation	112/72	1.10 (0.51-2.36)	0.807
Ordinal Goutallier per-grade model	Goutallier grade, per one-grade increase	112/72	1.43 (1.09-1.87)	0.010
Ordinal Goutallier per-grade model	Age, per year	112/72	0.98 (0.96-1.00)	0.094
Ordinal Goutallier per-grade model	Male sex	112/72	0.63 (0.36-1.11)	0.109
Ordinal Goutallier per-grade model	ECOG performance status	112/72	1.68 (1.28-2.21)	<0.001
Ordinal Goutallier per-grade model	Albumin, g/dL	112/72	0.93 (0.63-1.37)	0.697
Ordinal Goutallier per-grade model	Bilsky grade 3 compression	112/72	1.47 (0.84-2.57)	0.179
Ordinal Goutallier per-grade model	Katagiri moderate vs slow	112/72	4.24 (1.87-9.62)	<0.001
Ordinal Goutallier per-grade model	Katagiri rapid vs slow	112/72	4.60 (2.51-8.44)	<0.001
Low PVR primary model	Low PVR vs higher PVR	119/75	1.24 (0.73-2.10)	0.424
Low PVR primary model	Age, per year	119/75	0.99 (0.97-1.01)	0.490
Low PVR primary model	Male sex	119/75	0.57 (0.33-0.99)	0.046
Low PVR primary model	ECOG performance status	119/75	1.61 (1.23-2.10)	<0.001
Low PVR primary model	Albumin, g/dL	119/75	0.89 (0.59-1.33)	0.571
Low PVR primary model	Bilsky grade 3 compression	119/75	1.36 (0.78-2.37)	0.278
Low PVR primary model	Katagiri moderate vs slow	119/75	4.74 (2.07-10.83)	<0.001
Low PVR primary model	Katagiri rapid vs slow	119/75	5.28 (2.81-9.92)	<0.001
Low PMI primary model	Low PMI vs higher PMI	119/75	1.28 (0.76-2.15)	0.347
Low PMI primary model	Age, per year	119/75	0.99 (0.97-1.01)	0.511
Low PMI primary model	Male sex	119/75	0.57 (0.33-0.98)	0.043
Low PMI primary model	ECOG performance status	119/75	1.59 (1.21-2.08)	<0.001
Low PMI primary model	Albumin, g/dL	119/75	0.90 (0.60-1.34)	0.600
Low PMI primary model	Bilsky grade 3 compression	119/75	1.36 (0.78-2.38)	0.280
Low PMI primary model	Katagiri moderate vs slow	119/75	4.75 (2.08-10.84)	<0.001
Low PMI primary model	Katagiri rapid vs slow	119/75	5.38 (2.89-10.02)	<0.001
Axial phenotype primary model	One abnormality vs neither	109/69	1.23 (0.60-2.55)	0.574
Axial phenotype primary model	Both abnormalities vs neither	109/69	2.64 (1.15-6.03)	0.021
Axial phenotype primary model	Age, per year	109/69	0.99 (0.96-1.01)	0.271
Axial phenotype primary model	Male sex	109/69	0.58 (0.33-1.02)	0.058
Axial phenotype primary model	ECOG performance status	109/69	1.60 (1.21-2.11)	<0.001
Axial phenotype primary model	Albumin, g/dL	109/69	0.92 (0.61-1.39)	0.685
Axial phenotype primary model	Bilsky grade 3 compression	109/69	1.55 (0.87-2.76)	0.140
Axial phenotype primary model	Katagiri moderate vs slow	109/69	4.29 (1.81-10.18)	<0.001
Axial phenotype primary model	Katagiri rapid vs slow	109/69	4.49 (2.37-8.49)	<0.001
Axial phenotype PNI/log-NLR model	One abnormality vs neither	109/69	1.51 (0.75-3.07)	0.250
Axial phenotype PNI/log-NLR model	Both abnormalities vs neither	109/69	3.15 (1.33-7.44)	0.009

Model	Term	n/events	HR (95% CI)	p value
Axial phenotype PNI/log-NLR model	Age, per year	109/69	0.99 (0.96-1.01)	0.251
Axial phenotype PNI/log-NLR model	Male sex	109/69	0.59 (0.33-1.04)	0.066
Axial phenotype PNI/log-NLR model	ECOG performance status	109/69	1.60 (1.22-2.09)	<0.001
Axial phenotype PNI/log-NLR model	PNI	109/69	1.00 (1.00-1.01)	0.203
Axial phenotype PNI/log-NLR model	Log-NLR	109/69	1.35 (0.95-1.91)	0.093
Axial phenotype PNI/log-NLR model	Bilsky grade 3 compression	109/69	1.49 (0.84-2.64)	0.172
Axial phenotype PNI/log-NLR model	Katagiri moderate vs slow	109/69	4.38 (1.80-10.63)	0.001
Axial phenotype PNI/log-NLR model	Katagiri rapid vs slow	109/69	4.63 (2.42-8.86)	<0.001
Axial phenotype surgical-strategy sensitivity model	One abnormality vs neither	109/69	1.31 (0.63-2.73)	0.472
Axial phenotype surgical-strategy sensitivity model	Both abnormalities vs neither	109/69	3.35 (1.39-8.07)	0.007
Axial phenotype surgical-strategy sensitivity model	Age, per year	109/69	0.99 (0.96-1.01)	0.215
Axial phenotype surgical-strategy sensitivity model	Male sex	109/69	0.59 (0.34-1.05)	0.071
Axial phenotype surgical-strategy sensitivity model	ECOG performance status	109/69	1.65 (1.24-2.20)	<0.001
Axial phenotype surgical-strategy sensitivity model	Albumin, g/dL	109/69	0.92 (0.61-1.39)	0.702
Axial phenotype surgical-strategy sensitivity model	Bilsky grade 3 compression	109/69	1.88 (1.00-3.54)	0.051
Axial phenotype surgical-strategy sensitivity model	Katagiri moderate vs slow	109/69	4.63 (1.85-11.59)	0.001
Axial phenotype surgical-strategy sensitivity model	Katagiri rapid vs slow	109/69	4.56 (2.42-8.59)	<0.001
Axial phenotype surgical-strategy sensitivity model	Decompression + short instrumentation vs decompression-only/augmentation	109/69	0.63 (0.30-1.32)	0.224
Axial phenotype surgical-strategy sensitivity model	Decompression + long instrumentation vs decompression-only/augmentation	109/69	0.59 (0.29-1.23)	0.160
Axial phenotype surgical-strategy sensitivity model	Corpectomy/360 reconstruction vs decompression-only/augmentation	109/69	1.02 (0.47-2.22)	0.957
Solid-tumor-only Goutallier model	Goutallier 3-4 vs 0-2	85/63	1.71 (0.96-3.06)	0.069
Solid-tumor-only Goutallier model	Age, per year	85/63	0.99 (0.96-1.01)	0.292
Solid-tumor-only Goutallier model	Male sex	85/63	0.57 (0.31-1.06)	0.077
Solid-tumor-only Goutallier model	ECOG performance status	85/63	1.46 (1.07-2.00)	0.017
Solid-tumor-only Goutallier model	Albumin, g/dL	85/63	0.86 (0.56-1.33)	0.495
Solid-tumor-only Goutallier model	Bilsky grade 3 compression	85/63	2.10 (1.08-4.10)	0.030
Solid-tumor-only Goutallier model	Katagiri moderate vs slow	85/63	2.82 (1.11-7.17)	0.030
Solid-tumor-only Goutallier model	Katagiri rapid vs slow	85/63	3.18 (1.46-6.90)	0.004
Solid-tumor-only axial phenotype model	One abnormality vs neither	83/61	1.27 (0.58-2.78)	0.542
Solid-tumor-only axial phenotype model	Both abnormalities vs neither	83/61	2.71 (1.07-6.85)	0.035
Solid-tumor-only axial phenotype model	Age, per year	83/61	0.99 (0.96-1.01)	0.352
Solid-tumor-only axial phenotype model	Male sex	83/61	0.65 (0.35-1.23)	0.188
Solid-tumor-only axial phenotype model	ECOG performance status	83/61	1.43 (1.03-1.97)	0.033
Solid-tumor-only axial phenotype model	Albumin, g/dL	83/61	0.91 (0.57-1.44)	0.677
Solid-tumor-only axial phenotype model	Bilsky grade 3 compression	83/61	2.32 (1.15-4.68)	0.019
Solid-tumor-only axial phenotype model	Katagiri moderate vs slow	83/61	2.77 (1.02-7.55)	0.046

Model	Term	n/events	HR (95% CI)	p value
Solid-tumor-only axial phenotype model	Katagiri rapid vs slow	83/61	2.78 (1.26-6.14)	0.011

Cox models report hazard ratios with 95% confidence intervals. Reference categories are indicated by contrast wording where applicable.

Supplementary Table S7. Full Firth logistic regression model coefficients.

Model	Term	n	OR (95% CI)	p value
90-day mortality: Goutallier model	Goutallier 3-4 vs 0-2	112	4.16 (1.29-15.16)	0.017
90-day mortality: Goutallier model	Age, per year	112	0.96 (0.91-1.02)	0.180
90-day mortality: Goutallier model	Male sex	112	1.01 (0.28-4.01)	0.983
90-day mortality: Goutallier model	ECOG performance status	112	1.50 (0.85-2.79)	0.165
90-day mortality: Goutallier model	Albumin, g/dL	112	0.56 (0.23-1.33)	0.188
90-day mortality: Goutallier model	Bilsky grade 3 compression	112	1.99 (0.50-8.21)	0.323
90-day mortality: Goutallier model	Katagiri moderate vs slow	112	2.97 (0.36-24.89)	0.298
90-day mortality: Goutallier model	Katagiri rapid vs slow	112	5.13 (1.32-29.56)	0.017
90-day mortality: phenotype model	One abnormality vs neither	109	2.63 (0.36-33.09)	0.358
90-day mortality: phenotype model	Both abnormalities vs neither	109	19.26 (2.69-274.47)	0.002
90-day mortality: phenotype model	Age, per year	109	0.96 (0.90-1.03)	0.236
90-day mortality: phenotype model	Male sex	109	1.52 (0.37-7.36)	0.566
90-day mortality: phenotype model	ECOG performance status	109	1.44 (0.77-2.86)	0.250
90-day mortality: phenotype model	Albumin, g/dL	109	0.78 (0.28-2.13)	0.625
90-day mortality: phenotype model	Bilsky grade 3 compression	109	2.52 (0.55-12.50)	0.230
90-day mortality: phenotype model	Katagiri moderate vs slow	109	4.58 (0.46-48.99)	0.186
90-day mortality: phenotype model	Katagiri rapid vs slow	109	4.55 (1.07-29.08)	0.040
Frankel improvement: Goutallier model	Goutallier 3-4 vs 0-2	66	0.28 (0.08-0.83)	0.022
Frankel improvement: Goutallier model	Age, per year	66	0.99 (0.94-1.04)	0.638
Frankel improvement: Goutallier model	Male sex	66	1.03 (0.32-3.36)	0.956
Frankel improvement: Goutallier model	ECOG performance status	66	0.47 (0.19-0.98)	0.042
Frankel improvement: Goutallier model	Bilsky grade 3 compression	66	1.76 (0.51-6.63)	0.372
Frankel improvement: Goutallier model	Katagiri moderate vs slow	66	0.54 (0.09-2.93)	0.476
Frankel improvement: Goutallier model	Katagiri rapid vs slow	66	0.82 (0.23-2.80)	0.749
Frankel improvement: Goutallier model	Preoperative Frankel grade	66	0.54 (0.22-1.23)	0.145
Frankel improvement: phenotype model	One abnormality vs neither	63	0.60 (0.15-2.26)	0.452
Frankel improvement: phenotype model	Both abnormalities vs neither	63	0.14 (0.02-0.68)	0.014
Frankel improvement: phenotype model	Age, per year	63	0.98 (0.92-1.02)	0.349
Frankel improvement: phenotype model	Male sex	63	1.01 (0.28-3.63)	0.982
Frankel improvement: phenotype model	ECOG performance status	63	0.54 (0.22-1.17)	0.122
Frankel improvement: phenotype model	Bilsky grade 3 compression	63	1.81 (0.50-7.36)	0.372
Frankel improvement: phenotype model	Katagiri moderate vs slow	63	0.77 (0.12-4.78)	0.772
Frankel improvement: phenotype model	Katagiri rapid vs slow	63	0.93 (0.26-3.36)	0.916
Frankel improvement: phenotype model	Preoperative Frankel grade	63	0.61 (0.24-1.41)	0.247

Firth logistic models report odds ratios with 95% confidence intervals. The 90-day mortality models adjusted for age, sex, ECOG performance status, albumin, Bilsky grade 3 compression, and Katagiri-style tumor-growth group. The Frankel-improvement models adjusted for age, sex, ECOG performance status, Bilsky grade 3 compression, Katagiri-style tumor-growth group, and preoperative Frankel grade.
